# Overheated and Understudied: A Scoping Review of Heat‐Related Health Impacts in the Arabian Peninsula

**DOI:** 10.1029/2024GH001277

**Published:** 2025-06-30

**Authors:** Anais Teyton, Jennifer Bailey, Eqi Luo, Rahaf Ajaj, Colin Raymond, Cascade Tuholske, Tarik Benmarhnia

**Affiliations:** ^1^ Herbert Wertheim School of Public Health and Human Longevity Science University of California San Diego CA USA; ^2^ School of Public Health San Diego State University San Diego CA USA; ^3^ Scripps Institution of Oceanography University of California San Diego CA USA; ^4^ Department of Earth Sciences Montana State University Bozeman MT USA; ^5^ College of Health Science Abu Dhabi University Abu Dhabi United Arab Emirates; ^6^ Joint Institute for Regional Earth System Science and Engineering University of California Los Angeles CA USA; ^7^ Geospatial Core Facility Montana State University Bozeman MT USA; ^8^ Irset Institut de Recherche en Santé Environnement et Travail UMR‐S 1085 Inserm University of Rennes EHESP Rennes France

**Keywords:** Arabian peninsula, morbidity, mortality, extreme heat, land cover, land use

## Abstract

Extreme heat is worsening due to climate change, and, in combination with increasing urban growth, is an escalating public health concern. In the Arabian Peninsula, the wet‐bulb temperature is projected to surpass theoretical human tolerance limits during the 21st century. Yet, heat research in the region has generally not focused on health impacts, and it is unclear how epidemiologic literature has investigated this. We performed a scoping review to examine the existing literature that assessed the relationship between extreme heat and health outcomes in the Arabian Peninsula, collecting papers published from 2010 to 2024 from three databases. We identified and extracted detailed information from a limited number of studies (*n* = 12). The greatest number of studies were conducted in Kuwait (*n* = 8), with others in Saudi Arabia (*n* = 4), and Qatar, Oman, Yemen, and the United Arab Emirates (*n* = 1 each). Average temperature was the most used exposure (*n* = 9) assessed at the daily level (*n* = 10), using one or several meteorological stations (*n* = 9) from a single city (*n* = 8). The outcome was predominantly daily‐level (*n* = 10) mortality (*n* = 9) assessed at an ecological scale (*n* = 10) as opposed to the individual scale. While most studies included confounders (*n* = 10), their selection was not always consistent with best practices. Most papers did not assess effect modification (*n* = 8), and none investigated modification by land‐cover and land‐use change on the heat‐health relationship. We provide future research recommendations based on our findings. Additional studies are critical to better understand the heat‐health relationship in the Arabian Peninsula, which can aid intervention implementation.

## Key Points

- A scoping review was conducted to assess extreme heat and health outcomes in the Arabian Peninsula (2010–2024)
- A limited number of studies were identified in the region (*n* = 12)
- Recommendations provided for future research–for example, region/population, exposure assessment, outcome selection, and analytical considerations

## 1 Introduction

The effects of extreme heat exposure are exacerbated due to intensifying climate change, causing a myriad of health issues to populations across the world (Bell et al., 2024; Horton et al., 2016; Intergovernmental Panel On Climate Change, 2023). These issues include but are not limited to direct health outcomes, such as heat exhaustion and heat stroke, as well as indirect health outcomes, such as exacerbations of cardiovascular and respiratory diseases, which can result in death (Ebi et al., 2021; Mora, Counsell et al., 2017). Literature examining the relationship between extreme heat and health has grown rapidly in recent years, motivated by the substantial, although notably underestimated, health burden of heat in line with the increase in frequency, duration, and intensity of heat events in many regions resulting from climate change (Horton et al., 2016; Marx et al., 2021; Mora, Dousset et al., 2017). Several studies have identified that particular regions are at risk of surpassing temperatures and humidity levels tolerable for human survival in the 21st century, including South Asia and the Persian/Arabian Gulf region (Coffel et al., 2018; Mora, Dousset et al., 2017; Pal & Eltahir, 2016; Powis et al., 2023; Raymond et al., 2020). Historically, a wet‐bulb temperature (a heat ‐stress metric) combining temperature and humidity of ∼35°C has been theorized as the maximum heat survivability threshold for sustained exposure; however, recent studies have identified a much lower human adaptability limit to extreme heat that varies across sub‐populations and in both dry and humid climates (Guzman‐Echavarria et al., 2024; Vanos et al., 2023; Vecellio et al., 2022, 2024). Such extremes have the potential to cause disastrous public health and societal consequences, particularly where there is high population density and other factors contributing to socioeconomic vulnerability (Im et al., 2017; IPCC, 2007; Vecellio et al., 2023; World Bank Group, n.d.‐b). Despite these regional challenges, most studies assessing heat exposure impacts on health have been conducted in North America, Europe, Australia, and China, geographical areas that are projected to be less burdened by heat in the future compared to others (Klingelhöfer et al., 2023; Mora, Dousset et al., 2017; Vanos et al., 2023).

**Figure 1:**
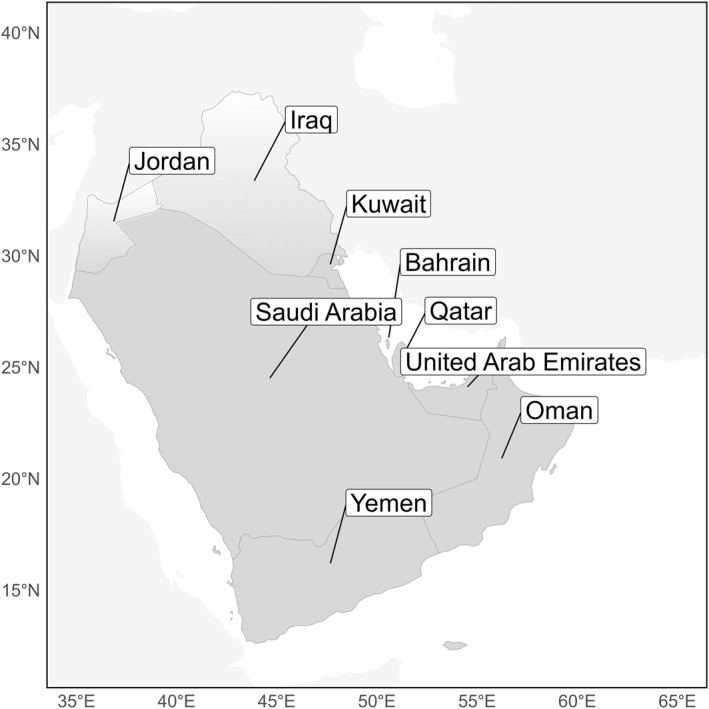
Map of countries located within the Arabian Peninsula region for the purposes of this study: Yemen, Oman, the United Arab Emirates, Qatar, Saudi Arabia, Bahrain, Kuwait, as well as southern Jordan and southern Iraq.

The Arabian Peninsula (Figure 1) is one such region projected to surpass survivable temperatures. However, this region has been critically understudied. One review conducted through May 2017 identified no papers examining the relationship between heat waves and morbidity and mortality in the Arabian Peninsula (Campbell et al., 2018). Similarly, another review investigating the impact of heat on mortality in low‐ and middle‐income countries identified no studies from 1980 to 2017 in this region (Green et al., 2019). Nonetheless, and regardless of its imminence, potentially unsurvivable, long‐duration heat stress has already been observed in the coastal Middle East (Raymond et al., 2020), and summertime air temperatures often exceed 45°C elsewhere in the region, such as Saudi Arabia, Kuwait, and Iraq (Powis et al., 2023; Raymond et al., 2020). Moreover, the wet‐bulb temperature has been found to often reach its maximum in the evening hours around the Southern Persian/Arabian Gulf, contrary to the usual assumption of greatest heat stress during the daytime (Raymond et al., 2024; Stull, 2011).

Furthermore, in the Middle East and North Africa (MENA) region, which includes the Arabian Peninsula, minimum temperatures have increased substantially in recent decades, adding to the challenging meteorological situation and complicating nighttime heat stress relief (Hamed et al., 2023). This extreme and, in some cases, humid heat is almost certainly intensified due to climate change. Several studies have found that large portions of the MENA population will be exposed to frequently recurring super‐ and ultra‐extreme heatwave events, with temperatures exceeding 56°C with durations extending to several weeks by the end of the century, noted as a conservative estimate for some locations (Christidis et al., 2023; Hajat et al., 2023; Zittis et al., 2021). Given these current and future temperature shifts and heat‐related health implications, more attention is urgently needed in this region to understand and reduce these health risks.

In the last five decades, rapid population growth and urbanization leading to drastic land‐cover and land‐use change have bolstered the increasing temperatures in the Arabian Peninsula (Menoret, 2014). The discovery of oil and subsequent “boom” in the early 1970s profoundly transformed much of the Arabian Peninsula (Menoret, 2014). The increase in oil revenues and related industrial development resulted in rapid economic expansion in Saudi Arabia and the Gulf States, encouraging domestic and international migration (Zimmer et al., 2024) and leading to more than half of the Arabian Peninsula population now being residents of urban areas (Alqurashi & Kumar, 2014; World Bank, 2023). Urban expansion, industrial activity, and other development initiatives have led to significant changes to the land‐use and land‐cover of this region (Alqurashi & Kumar, 2014), with large increases in impervious surfaces as well as irrigated vegetation in some areas.

Yet, research is limited regarding the complex relationship between climate dynamics and the urban heat island (UHI) effect in such regions as the Arabian Peninsula, dominated by desert and characterized by extreme heat, a scarcity of precipitation, and high evaporation rates of any available moisture (Alahmad, Tomasso et al., 2020; El Kenawy et al., 2024; Manoli et al., 2019; Powers et al., 1966; Rajeswari et al., 2024; L. Zhao et al., 2014). Global correlations and limited regional studies suggest that for dry heat, UHIs in the region are likely small and amenable to reduction by vegetation and albedo effects, while humid heat in urban areas poses a more stubborn challenge (Alahmad, Tomasso, et al., 2020; Manoli et al., 2019). Moreover, few studies have considered how land‐cover and land‐use changes may be modifying the heat‐health relationship in this region.

This rapid urbanization, in combination with heat nearing or surpassing human tolerance, will lead to severe health effects for local populations, especially for vulnerable groups with less coping capacity. In the past century, the MENA region has experienced one of the highest rates of population growth of any region globally, leading to demographic shifts and heat‐related vulnerability of migrant populations and outdoor workers (e.g., adverse health impacts and productivity loss) (Ewers et al., 2020; Mehmood et al., 2018; Yüceşahin & Tulga, 2017). Devastating heat‐related health consequences have been experienced recently, revealing how certain sub‐populations are at greater risk. For instance, in the summer of 2024, Hajj—an annual Islamic pilgrimage to Mecca, Saudi Arabia—occurred during a severe heat wave that led to over 1,300 deaths across a few days, where temperatures surpassed 113°F, or 45°C (Batrawy, 2024; Matthews et al., 2024). In previous years when Hajj has taken place in the summer, a large health burden has resulted from these high temperatures, physical exertion, and limited shade, despite preparedness and adaptation efforts put in place by the Saudi government (Ahmed et al., 2006). This is of particular concern given that over 2 million pilgrims participate in Hajj, many of whom are unacclimated to this extreme heat (Yezli et al., 2023a, 2024). Heat‐related health impacts have also taken place in the region during other religious and social mass gatherings (Alqahtani et al., 2021; Matzarakis & Fröhlich, 2015; Sofotasiou et al., 2015). Furthermore, occupational heat‐related injuries have occurred for workers within the region, as limited heat adaptation strategies for workers are imposed (Ioannou et al., 2021). A large proportion of the work force in certain countries within the Arabian Peninsula are migrant workers who have been subjected to poor living and working conditions, aggravating their heat vulnerability (Diop et al., 2020; Ewers et al., 2020; Iskander, 2021). Thus, susceptible sub‐populations and geographies should be considered when investigating the relationship between heat and health to assuage this inequitable burden.

As a result of these gaps in the literature and pressing public health concerns, we sought to conduct a scoping review to gather and examine existing epidemiological studies that assessed the relationship between heat exposure and adverse health outcomes in the Arabian Peninsula. We additionally provide recommendations for future studies based on the findings from our review.

## 2 Methods

### 2.1 Search Strategy and Eligibility Criteria

We aimed to identify recent epidemiological studies that assessed heat exposure impacts on human health in the Arabian Peninsula. This review followed the Preferred Reporting Items for Systematic Reviews and Meta‐Analyses' reporting guidelines for scoping reviews (PRISMA‐ScR) (Tricco et al., 2018). While not registered, our review protocol can be found in (Text S1 in Supporting Information S1). We selected the following three databases to identify peer‐reviewed research studies: Scopus, PubMed, and Web of Science. The search was limited to research articles (i.e., no commentaries, no reviews, etc.) published in the English language from 1 January 2010, to 10 October 2023, the date on which this initial search was conducted. A subsequent search was conducted on 20 January 2025, to update and complete this search from 1 January 2023 through 31 December 2024. These eligibility criteria were selected so as to include recent original articles that performed quantitative analyses on this heat‐health relationship in the region. Beyond these requirements, the title or abstract needed to include search terms from each of the following three groupings: (a) Exposure, (b) Outcome, and (c) Region. Text S1 in Supporting Information S1 provides the general and database‐specific search terms in each of these groupings. Specifically, terms commonly used in the epidemiologic literature assessing heat and health were selected for the exposure and outcome groupings, and both region‐ and country‐specific terms were incorporated to collect studies conducted in our area of interest.

### 2.2 Screening and Study Selection

In total, our initial search yielded 520 articles in Scopus, 251 articles in PubMed, and 419 in Web of Science. After removing duplicate articles, a total of 654 articles were identified. We identified 267 papers in the subsequent search for 2023–2024, including 214 from Scopus, 88 from PubMed, and 167 from Web of Science. Two reviewers (A.T. and J.B.) screened the titles and abstracts of these articles independently and assigned one of three categories to the articles: relevant (meaning the article assessed both the exposure and outcome of interest within the region), partially relevant (meaning the article assessed either the exposure or the outcome of interest within the region), and irrelevant (meaning the article assessed neither the exposure nor the outcome, and/or was not in the region of interest). The reviewers then compared their independent screening results and discussed all articles with different categories until a consensus was reached. A total of 74 articles were identified as “Relevant.”

The same two reviewers (A.T. and J.B.) then assessed these relevant articles in full to check that they met eligibility criteria. Studies were selected that assessed objectively measured chronic health outcomes, direct heat‐related health impacts, hospitalizations, or mortality (i.e., no infectious, vector‐, water‐, or food‐borne disease outcomes) as well as objectively measured temperature exposures (i.e., no subjective participant perception of temperature exposure). Moreover, papers that had other issues with the exposure or outcome, such as assessing an intervention as opposed to a temperature exposure or using derived rather than measured health outcomes, were removed. Lastly, papers that conducted in‐depth or advanced epidemiological analyses (i.e., omitting basic descriptive analyses such as solely conducting one‐ or two‐way tables or assessing correlation coefficients) were included in the review.

### 2.3 Data Extraction

A data extraction table was created by the two reviewers (A.T. and J.B.). These reviewers independently extracted these data, discussed all results and resolved any inconsistencies, and collaborated to create a finalized data extraction table. Key information from each study was extracted regarding the study characteristics (location, study design, time period assessed, type of analysis, statistical design, sample size, and sampling strategy), temperature exposure measurement (selected temperature metric, measurement source, and temporal and spatial scale of temperature exposure), health outcome measurement (health outcome assessed, and temporal and spatial scale of health outcome), analytical specifications and considerations (confounder and effect modification considerations as well as the assessment of landscape and urban form), and results (total and effect modification effect estimates).

We mapped the country of interest for each study and tabulated the selected temperature exposure and adverse health outcome by country. We then provided descriptive statistics grouped by study description and design, temperature exposure, health outcome, confounder and effect modification assessment, and analytical considerations and effect estimates.

### 2.4 Sensitivity Analysis

Given that our primary search included country‐ and region‐specific search terms, it was not likely to capture global‐scale analyses. A sensitivity analysis was therefore conducted specifically to retrieve relevant global studies from the Multi‐Country Multi‐City (MCC) Collaborative Research Network (London School of Hygiene & Tropical Medicine, n.d.‐a). Research produced within the MCC network includes studies assessing the relationship between heat exposure and adverse health outcomes using data assembled from hundreds of locations within a multitude of countries, including those in the Arabian Peninsula. Of the 54 available MCC studies screened by both reviewers (A.T. and J.B.) using the same criteria as the primary search on 18 April 2024, a total of 9 studies were found to be “Relevant.” A subsequent search was conducted on 20 January 2025, where, of the 19 more recent MCC studies published through 31 December 2024 that were screened by both reviewers (A.T. and J.B.) using the same criteria as the primary search, an additional 8 studies were found to be “Relevant.”

## 3 Results

### 3.1 Primary Search

**Figure 2:**
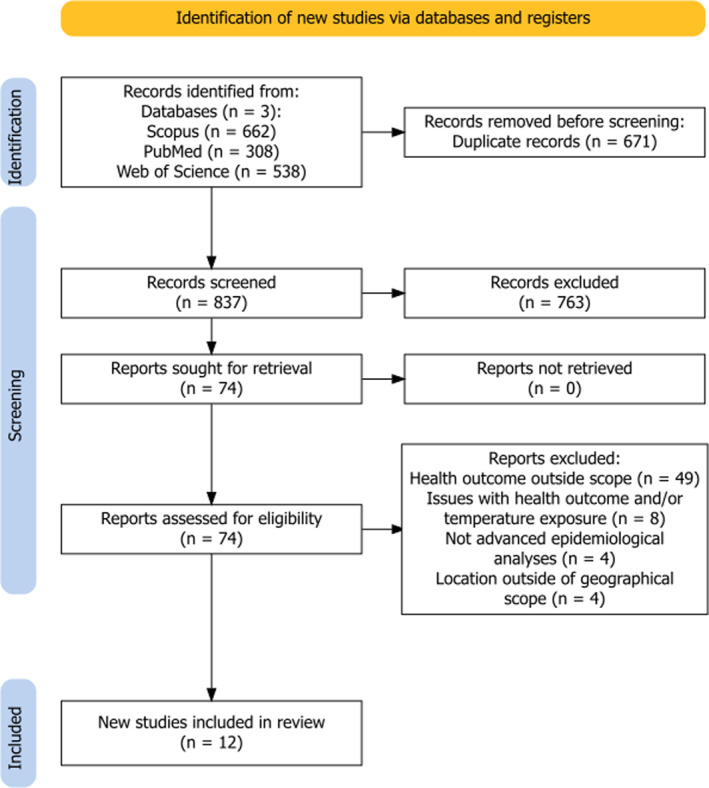
PRISMA flow diagram of scoping review selection and extraction (Haddaway et al., 2022). Details regarding inclusion and exclusion criteria are provided. To note: Reports removed for one or several reasons. A total of 12 studies were included in the review.

**Figure 3:**
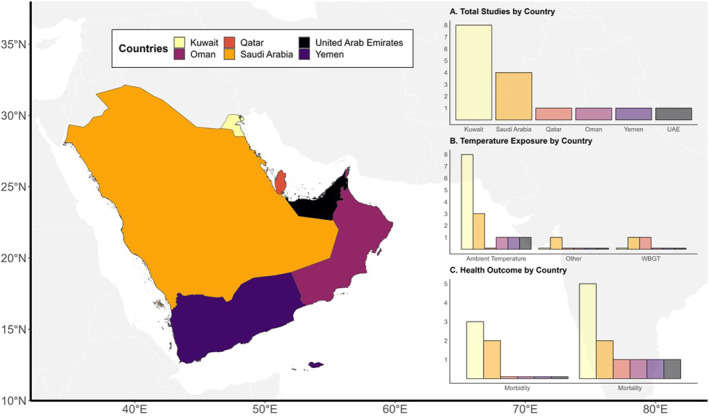
Map depicting the Arabian Peninsula with total included studies (*n* = 12) by country. Information is provided by country on temperature exposures and health outcomes used in all included studies.

After screening the 837 unique articles (excluding overlap) and examining the 74 relevant articles for eligibility, a total of 12 research articles were selected for this review. A flowchart depicting the inclusion and exclusion criteria for study selection is provided in Figure 2. Figure 3 provides a map of the distribution of the 12 studies identified in the primary search. These studies were published between 2019 and 2024, with most located in Kuwait (*n* = 8), Saudi Arabia (*n* = 4), and Qatar, Oman, Yemen, and the United Arab Emirates (*n* = 1 per country). Most studies utilized a longitudinal design (*n* = 10), although two opted for a retrospective or prospective cohort design. Apart from one study, every study included all health events as their sampling strategy (*n* = 11).

**Figure 4:**
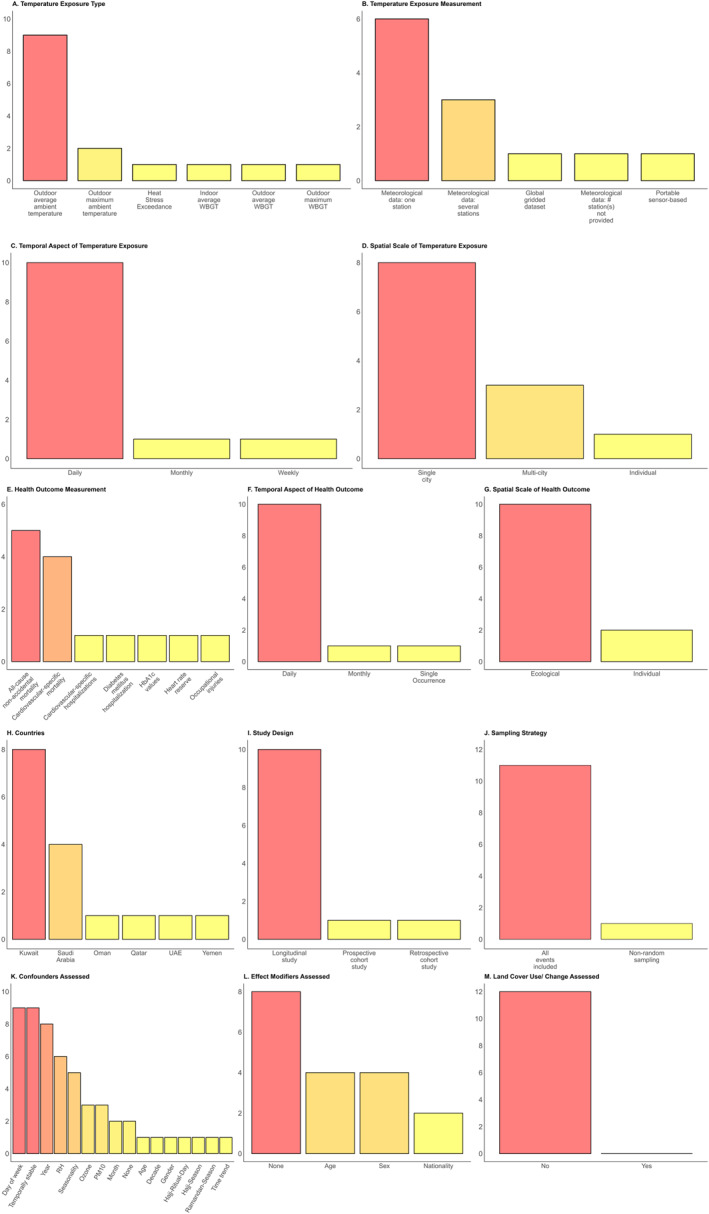
The assessed temperature exposure (a–d, first and second rows), health outcome (e–g, third row), study details (h–j, fourth row), and analytical considerations (k–m, fifth row) for the 12 studies included in the review. For the spatial scale of the adverse health outcome (g), the individual scale refers to data collected from single individuals, while the ecological scale refers to data aggregated at the population‐level instead. Note: For effect modifiers assessed (l), three of the four studies assessed effect modification by sex, while one assessed modification by gender. Colors ranging from yellow to red indicate a greater number of studies.

Table 1 summarizes the extraction results, and Figure 4 visualizes the results related to the temperature exposure, adverse health outcomes, study details, and analytical considerations (see the full extraction in, Table S1 in Supporting Information S1). A range of different temperature exposure metrics were used, however most studies utilized outdoor average ambient temperature (*n* = 9). Most studies measured temperature exposure using meteorological data (*n* = 9), with six studies using only one meteorological station (*n* = 4), three using several stations (*n* = 3), and one not stating the number of stations used (*n* = 1). A majority of the studies assessed a daily duration of temperature exposure (*n* = 10). Lastly, a majority of the studies assessed the temperature exposure at a single city‐level (*n* = 8).

**Table 1:** Summary Extraction Table for the Primary Search (*n* = 12)

Author (Year)	Country	Period	Study Design	Sample Size	Temperature (*T*) Exposure	Health Outcome	Effect Estimate	Confounders	Effect Modification
Alahmad et al. (2019)	Kuwait	2010–2016	Longitudinal (Time‐series)	Entire population included	Outdoor average temperature	All‐cause non‐accidental mortality	42.7°C (99th percentile T) versus optimum T (66th percentile, 34.7°C): RR = 1.63; 95% CI: 1.09 to 2.46 41.6°C (97.5th percentile T) versus optimum T: RR = 1.41; 95% CI: 1.02 to 1.93	Ozone, PM10, RH, year, and day of the week. Temporally stable confounders adjusted for by design.	None
				33,574 all‐cause non‐accidental deaths					
Pradhan et al. (2019)	Qatar	2009–2017	Longitudinal (Time‐series)	All Nepali migrant workers	Outdoor maximum wet‐bulb globe temperature (WBGT; “in‐shade” as indoors or full shade, without solar or other heat radiation)	Cardiovascular‐specific mortality	Cardiovascular deaths per 100,000 NMW: 1‐Month WBGT (in‐shade): *β* = 5.5 3‐Month WBGT (in‐shade): *β* = 5.7 95% CIs not explicitly stated	None	None
				over 1,300 Nepali migrant workers (NMW) died during study period					
Alahmad, Khraishah et al. (2020)	Kuwait	2010–2016	Longitudinal (Time‐series)	Entire population included	Outdoor average temperature	Cardiovascular‐specific mortality	42.7°C (99th percentile T) versus MMT (34.7°C): RR = 3.09; 95% CI: 1.72 to 5.55	Ozone, PM10, RH, year, seasonality, and day of the week. Temporally stable confounders adjusted for by design.	Age: Aged 15–64 years: RR = 3.84; 95% CI: 1.57 to 7.70 Aged above 65: RR = 2.29; 95% CI: 0.96 to 5.48 Sex: Males: RR = 3.53; 95% CI: 1.74–7.16 Females: RR = 2.36; 95% CI: 0.83 to 6.66
				15,609 total cardiovascular‐related deaths					
Alahmad, Shakarchi et al. (2020)	Kuwait	2010–2016	Longitudinal (Time‐series)	Entire population included	Outdoor average temperature	All‐cause non‐accidental and cardiovascular mortality	42.7°C (99th percentile T) versus MMT (66th percentile, 34.7°C) for non‐accidental mortality: RR = 1.63; 95% CI: 1.09 to 2.46 42.7°C versus MMT for cardiovascular‐specific mortality: RR = 3.09; 95% CI: 1.72 to 5.55	Ozone, PM10, RH, year, and day of the week. Temporally stable confounders adjusted for by design.	Total non‐accidental mortality (sex, age, and nationality): Males: RR = 2.08; 95% CI: 1.23 to 3.52 Females: RR = 1.14; 95% CI: 0.60 to 2.18 0–14 years old: RR = 0.29; 95% CI: 0.09 to 0.97 15–64 years old: RR = 2.28; 95% CI: 1.24 to 4.20 65+ years old: RR = 1.86; 95% CI: 1.02 to 3.39 Kuwaiti: RR = 1.38; 95% CI: 0.80 to 2.41 Non‐Kuwaiti: RR = 1.96; 95% CI: 1.10 to 3.52 Cardiovascular mortality (sex, age, and nationality): Males: RR = 3.53; 95% CI: 1.74 to 7.16 Females: RR = 2.36; 95% CI: 0.83 to 6.66 0–14 years old: omitted due to wide SEs 15–64 years old: RR = 3.84; 95% CI: 1.57 to 7.70 65+ years old: RR = 2.29; 95% CI: 0.96 to 5.48 Kuwaiti: RR = 2.98; 95% CI: 1.23 to 7.20 to Non‐Kuwaiti: RR = 3.15; 95% CI: 1.44 to 6.91
				33,472 all‐cause non‐accidental deaths					
Al‐Bouwarthan et al. (2020)	Saudi Arabia	2016 (June ‐ September)	Prospective cohort study	23 construction workers	Indoor and outdoor average WBGT	Heart Rate Reserve (HRR, as a measure of cardiovascular strain, %)	WBGT: *β* = 0.78; 95% CI: 0.45 to 1.12 HSE: *β* = 0.70; 95% CI: 0.36 to 1.04	None	None
					Heat Stress Exceedence (HSE)				
Alghamdi et al. (2021)	Saudi Arabia	15 November 2012–9 September 2018	Retrospecitve cohort study	168,614 adult Saudi patients at the Security Forces Hospital in Riyadh who had HbA1c analyzed	Outdoor maximum temperature	HbA1c values (%) divided into 2 categories: HbA1c < 7% (good glycemic control) and HbA1c ≥ 7% (poor glycemic control)	HbA1c ≥ 7% (poor glycemic control): High T (>35.8°C) versus low T (<26.1°C): OR = 1.134; 95% CI: 1.107 to 1.162 Moderate T (26.1°C–35.8°C) versus low T (<26.1°C): OR = 1.034; 95% CI: 1.007 to 1.062	Age and gender	None
					Categorical outdoor maximum temperature (high (>35.8°C), moderate (26.1°C–35.8°C), and low (<26.1°C))				
Alahmad et al. (2022)	Kuwait	2000–2016	Longitudinal (Time‐series and projection)	Entire population included	Outdoor average temperature	All‐cause non‐accidental and cardiovascular mortality	Non‐linear U‐shaped temperature‐mortality relationships identifed, but effect estimates not explicitly stated.	RH, year, seasonality, and day of the week. Temporally stable confounders adjusted for by design.	Age (0–65 and 65+), gender (men and women), and nationality (Kuwaitis and Non‐Kuwaitis). Those aged above 65 years and non‐Kuwaitis found to be at increased risk. Non‐linear, predominantly U‐shaped temperature‐mortality relationships identified, but effect estimates not explicitly stated.
				73,748 all‐cause non‐accidental deaths and 35,285 cardiovascular deaths					
Alahmad, Al‐Hemoud et al. (2023)	Kuwait	2015–2019 (1 June ‐ 31 August)	Longitudinal (Time‐series)	All private sector workers in Kuwait	Outdoor average temperature	Occupational injuries	39.4°C versus 37.0°C (10th percentile T): RR = 1.44; 95% CI: 1.34 to 1.53 40°C versus 37.0°C: RR = 1.48; 95% CI: 1.39 to 1.59 41°C versus 37.0°C: RR = 1.44; 95% CI: 1.27 to 1.63 42°C (90th percentile T) versus 37.0°C: RR = 1.21; 95% CI: 0.93 to 1.57	RH, year, seasonality, and day of the week. Temporally stable confounders adjusted for by design.	None
				3,710 occupational injuries					
Yezli et al. (2023b)	Saudi Arabia	1 February 2006–1 October 2015	Longitudinal (Time‐series)	Population of Mecca, Saudi Arabia	Outdoor average temperature	All‐cause non‐accidental mortality	Non‐linear U‐shaped temperature‐mortality relationship identified, but effect estimates not explicitly stated.	Year, Hajj‐Season, Ramadan‐Season, month, day of the week, and Hajj‐Ritual‐Day. Temporally stable confounders adjusted for by design.	None
				37,178 non‐accidental deaths			38°C (97.5th percentile T) versus MMT (48th percentile, 31.8°C): Attributable Mortality = 5.6%; 95% CI: −3.8 to 13.2		
Alahmad, Ali, et al. (2024)	Kuwait	2010–2019 (June ‐ August)	Longitudinal (Case‐crossover)	All individuals with unplanned hospital admissions for diabetes mellitus	Outdoor average temperature	Diabetes mellitus hospitalization	34°C versus 33°C (lowest summer T, rounded to the whole number): RR = 1.11; 95% CI: 1.01 to 1.23 38°C versus 33°C: RR = 1.58; 95% CI: 1.03 to 2.42 44°C versus 33°C: RR = 1.52; 95% CI: 0.74 to 3.13 Identified a non‐linear relationship between heat and diabetic hospitalizations during summer months (see RR estimates above). Excess diabetic admissions attributed to hot days above 33°C each year = 282; 95% CI: −14 to 473	Year, month, and day of the week. Temporally stable confounders adjusted for by design.	34°C versus 33°C (lowest summer T) (sex and age): Male: RR = 1.13; 95% CI: 0.99 to 1.29 Female: RR = 1.09; 95% CI: 0.95 to 1.26 Elderly (65+): RR = 1.10; 95% CI: 0.90 to 1.35 38°C versus 33°C (sex and age): Male: RR = 1.62; 95% CI: 0.91 to 2.88 Female: RR = 1.50; 95% CI: 0.81 to 2.78 Elderly (65+): RR = 1.58; 95% CI: 0.65 to 3.84 44°C versus 33°C (sex and age): Male: RR = 1.40; 95% CI: 0.52 to 3.78 Female: RR = 1.53; 95% CI: 0.55 to 4.26 Elderly (65+): RR = 2.39; 95% CI: 0.58 to 9.86
				8,960 unplanned hosptalizations					
Alwadi et al. (2024)	Kuwait	2010–2019	Longitudinal (Time‐series)	Entire population included	Outdoor average temperature	Cardiovascular‐specific hospital admissions for all cardiovascular diseases, ischemic heart disease, and stroke	All cardiovascular hospitalizations: 41°C versus MMT (20.7°C): RR = 1.292; 95% CI: 1.051 to 1.589 42°C versus MMT: RR = 1.309; 95% CI: 1.036 to 1.654 43°C versus MMT: RR = 1.326; 95% CI: 1.006 to 1.747 Attributable admissions for all hot days above the MMT = 20,569; 95% CI: 3,128 to 35,757 Ischemic heart disease hospitalizations: 41°C versus MMT (18.4°C): RR = 1.378; 95% CI: 1.046 to 1.815 42°C versus MMT: RR = 1.433; 95% CI: 1.052 to 1.951 43°C versus MMT: RR = 1.495; 95% CI: 1.042 to 2.144 Attributable admissions for all hot days above the MMT = 13,741; 95% CI: −1,941 to 26,169 Stroke hospitalizations: 41°C versus MMT (21.7°C): RR = 1.212; 95% CI: 0.780 to 1.882 42°C versus MMT: RR = 1.219; 95% CI: 0.756 to 1.965 43°C versus MMT: RR = 1.225; 95% CI: 0.730 to 2.056 Attributable admissions for all hot days above the MMT = 2,379; 95% CI: −2,533 to 6,287 Non‐linear, somewhat U‐shaped temperature‐hospitalization relationships identified.	RH, seasonality, time trends (not explicitly stated), and day of the week. Temporally stable confounders adjusted for by design.	None
				263,182 cardiovascular hospitalizations					
Zhao et al. (2024)	Kuwait, Oman, Saudi Arabia, United Arab Emirates, Yemen	Kuwait: 2000–2016 (warm season) Other countries: Not explicitly stated	Longitudinal (Time‐series)	Entire population included	Outdoor average temperature	All‐cause and/or non‐external mortality (not specified at the country‐level)	Effect estimates not explicity provided at the country‐level.	Decade, year, seasonality, and day of the week. Temporally stable confounders adjusted for by design.	None
				Deaths: N/A	heat wave: daily mean temperature ≥95th percentiles of year‐round temperature range with duration ≥2 days				

*Note.* Additional details are provided in Table S1 in Supporting Information S1. *T* = temperature; RR = risk ratio; PM10 = particulate matter ≤10 μm; RH = relative humidity; NMW = Nepali migrant workers; WBGT = wet‐bulb globe temperature; MMT = minimum mortality temperature; SE = standard error; HSE = heat stress exceedence; HRR = Heart Rate Reserve; *β* = beta estimates; OR = odds ratio.

Regarding the health outcome of interest, most articles focused on either cardiovascular‐specific mortality (*n* = 4) or all‐cause non‐accidental mortality (*n* = 5). Other health outcomes assessed included heart rate reserve, occupational injuries, HbA1c values, diabetes mellitus hospitalization, and cardiovascular‐specific hospitalization. Most studies also assessed the health outcome of interest at the daily level (*n* = 10). Seven studies assessed the health outcome at the ecological scale (*n* = 10), compared to two studies which did so at the individual scale (*n* = 2).

Only two studies did not account for any confounders within their models (*n* = 2). Of those that included confounders, the most frequently included confounders were temporally stable confounders by design (*n* = 9), various time trends (*n* = 9), relative humidity (RH) (*n* = 6), PM_10_ (*n* = 3), and ozone (*n* = 3). Age and gender were each accounted for as confounders once (*n* = 1). In contrast, most studies did not assess for effect modification (*n* = 8). Age and sex/gender were the most frequently assessed effect modifiers (*n* = 4), followed by nationality (*n* = 2). None of the included studies considered possible modification of the heat‐health relationship by landscape and urban form.

With the exception of one paper that utilized mixed methods, all other papers conducted quantitative analyses (additional details regarding the specific methodology used in each study are outlined in Table S1 in Supporting Information S1). Two studies (*n* = 2) did not include explicit risk ratio values, as they investigated the temperature‐health relationships non‐linearly and opted to provide temperature‐health curves instead. Both papers identified U‐shaped relationships, with health risks rising exponentially with increasingly warmer temperatures. One study provided neither effect estimates nor temperature‐health relationship curves. All studies identified positive associations between the temperature exposure metric and selected adverse health outcomes. Risk ratios ranged from 1.11 (95% CI: 1.01, 1.23) to 3.09 (95% CI: 1.72, 5.55), beta coefficients ranged from 0.70 (95% CI: 0.36, 1.04) to 5.7 (95% CI not explicitly provided), and odds ratios ranged from 1.03 (95% CI: 1.01, 1.06) to 1.13 (95% CI: 1.11, 1.16). Across studies that assessed effect modification, males tended to be more susceptible than women and non‐nationals more susceptible than nationals. Differences in effect estimates by age were noted, where two papers identified that those 15–65 years old were at greater risk than their counterparts, whereas one paper identified that those greater than 65 years old were at greatest risk; however, it is important to note that both of these sub‐populations were at increased risk from being exposed to higher temperatures.

### 3.2 Sensitivity Analysis

Extraction information can be found for the 12 studies collected from the sensitivity analysis in (Table S2 in Supporting Information S1) (Chen et al., 2024; Hundessa et al., 2024; Lo et al., 2023; Mistry et al., 2022; Rai et al., 2023; Vicedo‐Cabrera et al., 2021; Yang et al., 2024; Zhao et al., 2021). All of these studies focused on Kuwait and used a longitudinal design, where all events were included. Moreover, all papers assessed the heat‐health relationship from 2000 to 2016 except for one paper that examined 2000–2014, with three papers explicitly focusing on the warm season. Ten analyses used a two‐stage analytical design, while two analyses used a three‐stage analytical design. For the temperature exposure metric, 11 studies utilized outdoor average temperature. Five papers aggregated their temperature exposure metric from global gridded data, and eight papers utilized weather station data predominantly from two sources; however, the number of weather stations used was not explicitly provided. All papers assessed the temperature exposure metric at the daily level for a single city, and, with the exception of two studies, extrapolated to the country‐level. Eight papers focused on non‐external or all‐cause mortality, while four papers selected cardiovascular‐specific mortality, and one paper selected respiratory‐specific mortality. All papers assessed their mortality outcome at the ecological scale at the daily level. Six papers assessed for non‐linear relationships through exposure‐response function curves and did not provide specific effect estimates as a result; these articles tended to identify a U‐shaped heat‐health relationship. We present the first stage effect estimates (where provided) from these studies, as these assess the heat‐health relationship with observed data for the country or city of interest. Positive effect estimates were predominantly identified across these papers. All studies adjusted on time‐varying confounders (e.g., day of the week, month, seasonality, year, and long‐term time trends). Two papers assessed for effect modification, where one assessed the relationship by age and the other by air pollution exposure (e.g., PM_10_ and O_3_). Older adults (those greater than 74 years old, followed by those 65–74 years old) were also found to be more susceptible to the risk of mortality from increasing temperature exposure, and exposure to high air pollution days tended to lead to a stronger risk in adverse health outcomes from increasing heat. Again, no papers assessed landscape or urban form impacts on the heat‐health relationship. However, one study investigated the influence of land use land‐cover change scenarios on the temperature‐health relationship at the sub‐continental level using future projections.

## 4 Discussion

**Figure 5:**
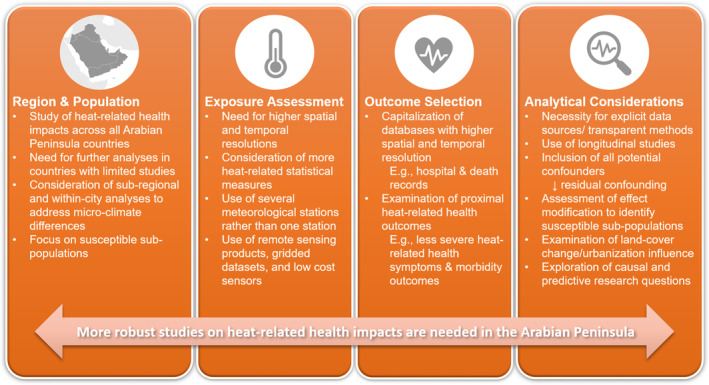
Recommendations based on the scoping review on heat and health in the Arabian Peninsula (*n* = 12 studies).

We aimed to explore studies published between 2010 and 2024 that examined heat impacts on health in the Arabian Peninsula. We identified only 12 studies that have done so (the first one being from 2019), most of which were conducted in Kuwait. Daily outdoor average ambient air temperature tended to be the most utilized exposure metric, and a majority of studies assessed all‐cause non‐accidental or cardiovascular‐specific mortality. It is clear that studies with differing geographic and sub‐population focus areas are needed to elucidate and examine the heat‐health relationship in the Arabian Peninsula. The lack of studies in this area is particularly troubling, given that the Arabian Peninsula will be one of the most impacted regions globally in the coming decades due to temperatures surpassing human tolerance and that the temperatures assessed as minimum mortality temperatures or in relation to health outcome estimates in most studies already surpass the human heat threshold for continuous exposure as it is. This highlights the urgent need for additional studies that supplement the existing research for this region, also investigating a range of health impacts from extreme heat exposures in different parts of the region and identifying susceptible populations using robust methods. Below, we highlight the four primary issues we identified from the existing literature and provide recommendations for future research (Figure 5): (a), Region and Population, (b) Exposure Assessment, (c) Outcome Selection, and (d) Analytical Considerations.

### 4.1 Region and Population

Kuwait and Saudi Arabia were most studied, with Qatar, Oman, Yemen, and the United Arab Emirates assessed once each, leaving most countries in the Arabian Peninsula. with poor or no coverage. The paucity of studies highlights the significant knowledge gap in the literature of local heat‐health relationship nuances in this region, especially as coastal and inland populations within these countries experience different types of heat (e.g., humid and dry, respectively) (Akasha et al., 2023; Raymond et al., 2024).

Kuwait, the focus of much of the existing work, consists of an entirely urban population, with migrants constituting over 70% of the total population in the country. Many migrants partake in physically demanding and oftentimes outdoor jobs, increasing the risk of heat exposure and adverse health outcomes (Mehmood et al., 2018; World Bank Group, n.d.‐a). Yet only one study in Kuwait explicitly investigated occupational injuries related to heat, and it focused solely on private sector workers and did not include government employees, domestic workers, and informal economy workers. Of the eight studies conducted in Kuwait, only two examined how the heat‐health relationship differed between Kuwaiti and non‐Kuwaiti populations, noting a higher risk of the outcome for non‐Kuwaitis. Similarly, Qatar, with its almost entirely urbanized population, 75% of whom are migrants, had one study meet the inclusion criteria, which specifically focused on Nepali migrant workers in Doha (World Bank Group, n.d.‐a). Understanding how heat affects workers' health and, consequently, productivity, is especially relevant to environmental justice and economically significant for the Arabian Peninsula, given the large expatriate workforce in the region. By some global estimates, Qatar, Bahrain, and the United Arab Emirates are the top 3 countries for per‐capita annual labor‐productivity loss due to humid heat (Parsons et al., 2022). Saudi Arabia greatly surpasses both Kuwait and Qatar in terms of land area and population, and there exists a pressing need to examine the impact of heat on Saudi residents' and visitors' health. However, just three studies were conducted in Saudi Arabia, which focus on susceptible sub‐populations in highly constrained study areas (e.g., a construction site, a hospital, and the city of Mecca) (Almulhim & Cobbinah, 2023; World Bank Group, n.d.‐c), making these findings difficult to generalize. There remains a crucial need to continue to assess how the heat‐health relationship varies within countries of this region, especially among vulnerable sub‐populations (e.g., expanding older population, migrant workers, and those of lower socioeconomic status) that have thus far been minimally assessed or not at all (Almulhim & Cobbinah, 2023).

Subregional climate differences have contributed to heterogeneity in the way land‐cover and land‐use change affects temperature and humidity within the Arabian Peninsula, making it especially important to conduct these studies at a smaller geographical scale where data permits (Aina et al., 2017; Elhacham & Alpert, 2021; Rahman et al., 2017). Some cities of the Arabian Peninsula experience an “urban cool island,” where urban areas have been observed to be cooler than rural surroundings during the day due to shading from buildings and evapotranspiration from vegetation; these introduced features also impede the progress of the sea breeze, with a net effect on heat stress that varies based on time, location, and exposure characteristics (Al‐Ruzouq et al., 2022; Lazzarini et al., 2013; Safieddine et al., 2022). Urban land‐surface temperatures in the region are nearly always higher than rural ones (Aina et al., 2017; Alqurashi & Kumar, 2019; Elhacham & Alpert, 2021; Rahman et al., 2017), highlighting the importance of considering potential pattern differences between land‐surface and air temperature extremes (Hu et al., 2019). While studies have observed differential findings in terms of daytime surface temperature or UHI effects in urban areas, a trend of increasing nighttime or minimum temperatures has been relatively consistent across the literature in this region (Aina et al., 2017; Al‐Ruzouq et al., 2022; El Kenawy et al., 2024; Safieddine et al., 2022). The effect of humidity, especially in densely populated coastal cities and areas in proximity to irrigated spaces, is crucial to consider in the Arabian Peninsula as the wet‐bulb temperature has been found to be increasing, with especially high values in early evenings, times when people tend to go outside (Aina et al., 2017; Raymond et al., 2020, 2024; Safieddine et al., 2022).

The spatial heterogeneity of heat leads to varied and localized health risks across different urban and rural areas as well as climatic types. This is also a critical health equity issue, as susceptible sub‐populations living in countries in the region contribute minimally to climate change and benefit little from the extraordinary oil and gas exportations while being one of the most impacted areas in the world (Lara Ibarra et al., 2016; United Nations Economic and Social Commission for Western Asia, 2017). Intense and increasing heat across the Arabian Peninsula, combined with humidity near the Red Sea and Persian/Arabian Gulf, point toward a burgeoning public health challenge that needs to be further elucidated as it manifests differently across the region's diverse geographies and socioeconomic settings.

### 4.2 Exposure Assessment

Most studies focused on outdoor average ambient temperature; however, additional studies are needed to investigate the role of metrics such as wet‐bulb temperature and wet‐bulb globe temperature as well as heat variability, to better understand the role of humidity, other meteorological factors, and the range of high temperature experienced in the course of a day (including diurnal temperature variation), respectively. It is important to note that two studies assessed for temperature variability impacts on adverse health outcomes for countries in this region (Wen et al., 2024; Wu et al., 2022); however, these studies were not included, as they did not differentiate between hot and cold temperature extremes. Moreover, additional statistical measures could be explored, such as the maximum, minimum, or diurnal values, rather than a sole reliance on average measures (AlSarmi & Washington, 2011; Anderson & Bell, 2011; Baldwin et al., 2023; Zhang et al., 2014). This would allow for an improved characterization of heat in this region and aid in identifying which metrics would be most important to monitor, forecast, and devise mitigation/adaptation measures for health.

In our review, many studies focused on Kuwait City and utilized meteorological data averaged across a collection of stations in close proximity to Kuwait City along the coast. Even in Kuwait, this strategy may not adequately capture the spatial variation in localized micro‐heat exposure across all urban areas of the country (World Bank Group, 2023). Additionally, a few studies utilized weekly or monthly heat exposure, likely leading to imprecise exposure assignment (Lee et al., 2016). Other high spatial and temporal temperature data sources should be considered, such as from remote sensing instruments, gridded data sets, and low‐cost sensors (Bailey et al., 2020; Ceccato et al., 2010; Hooker et al., 2018; Li et al., 2023; Menne et al., 2012; Spangler et al., 2019; Vecellio & Vanos, 2024; Young et al., 2014). In addition, low‐cost sensors can be used indoors (Alshammari et al., 2023) or be worn by an individual, providing the possibility of examining indoor temperature exposure (as Al‐Bouwarthan and colleagues (2020) did with wet‐bulb globe temperature) as well as dynamic temperature exposure, minimizing the likelihood of exposure misclassification.

### 4.3 Outcome Selection

Studies predominantly assessed non‐accidental/non‐external all‐cause mortality or cardiovascular‐specific mortality as their outcome of interest. Additional cause‐specific mortality metrics should also be collected and investigated to better understand direct and indirect causes of death linked to heat exposure. More precise prevention strategies could then be implemented. Given the numerous proximal and less severe direct heat‐related health symptoms as well as the range of indirect heat‐related health impacts, adverse health outcomes other than mortality should also be studied (Dapi et al., 2010; Mora, Counsell et al., 2017; Teyton et al., 2021; Van Loenhout et al., 2016). Addressing these morbidity outcomes provides opportunities for earlier interventions.

While most papers predominantly assessed health outcomes at the daily level, two studies either measured the outcome during a single event or at the monthly level. Like the temperature exposure, a more refined temporal scale with repeated measures is critical to improve the understanding of this heat‐health relationship and reduce the risk of misclassification bias. While we did not include papers that investigated subjective or non‐validated health measures, we noted several studies that incorporated these suboptimal outcomes. Although the only option in most of the region due to a lack of data or limited data access, subjective outcome measures can lead to information bias, such as through participant recall, observer bias, and leading questions. Morbidity outcomes measured using objective medical devices, diagnostic instruments, or validated assessment scales as well as hospital and death records should be utilized where possible in such analyses instead to reduce the likelihood of bias. Through the findings of this review, we acknowledge that access to such data may be limited, and we highlight the value for authorities and scholars in the region to collaborate and focus on a wider range of health outcomes.

### 4.4 Analytical Considerations

While most studies focused on causal research questions, a few studies instead answered descriptive questions using simple statistical analyses. Robust predictive and causal research questions should be explored in the Arabian Peninsula, as the answers can help inform what may happen in the future and improve understanding of the etiology of the heat‐health relationship (Kamper, 2020). Additionally, many studies primarily focused on assessing the heat‐health relationship of interest up until 2016; however, this relationship needs to be quantified over more recent years, given the continuing urbanization with population increase, increasing extreme heat trends, and devastating mortality events that have since occurred (Labban et al., 2023; Syed et al., 2024; Vinodhkumar et al., 2024). Several studies also did not provide details regarding the data sources for the temperature exposure, the health outcomes nor some analytical choices (e.g., the choice of splines functions, knots, reference temperature, or choice of lagged exposures). The methodology in these studies must be clear and transparent for the sake of reproducibility and replicability.

Moreover, a quarter of the studies, which tended to be individual‐level studies, either adjusted on a minimal set of confounders or did not adjust for any confounders at all, which likely biases the relationship between temperature exposure and adverse health impacts. For these individual‐level studies, more demographic characteristics that influence both the exposure and outcome should be adjusted on, such as age, sex, nationality, and socioeconomic characteristics. Reliable demographic or health data sources, particularly at the individual level, would be especially helpful in this context as a means to reduce the possibility of residual confounding. Most papers included in our review used a time series design and adjusted for a robust set of time‐varying confounders, such as short‐term and long‐term time trends. Additionally, some of these studies adjusted for other environmental exposures, such as RH and air pollutants. However, it is not advised to adjust on such confounders when assessing temperature‐health relationships, as RH factors temperature into its metric and its use should be avoided compared to mass‐based humidity metrics, and temperature changes tends to influence air pollution levels, rather than the reverse (Baldwin et al., 2023; Buckley et al., 2014). These air pollutants, such as ozone, are instead mediators and should be analyzed accordingly (Alari et al., 2023; Reid et al., 2012). Thus, authors should be cautious and transparent of their assumptions when selecting potential confounders.

Moreover, effect measure modification was not considered by most studies. Age, sex, and nationality were considered by four studies, although these effect modifiers should be assessed in other countries and sub‐regions. This can provide insight regarding health inequities and sub‐populations that may be prioritized during extreme heat. Additional effect modifiers would also be valuable, such as socioeconomic characteristics, type of occupation, Hajj pilgrim status, access to heat‐related resources, climate zones or sub‐regions, and rural and urban classifications.

Lastly, land and urban development metrics should be assessed to better guide future research and can aid in mapping and understanding local aspects that influence heat exposure. These metrics may be calculated from raw or processed geospatial data derived from satellite science missions, airborne campaigns, geographic information systems, and street view images. With high spatiotemporal resolution, these often open‐source data can be used to estimate urban land expansion rates, conversion between different land covers, population density, three‐dimensional urban form, and urban activity development patterns (Asabere et al., 2020; Ekkel & De Vries, 2017; Güneralp et al., 2020; Jarvis et al., 2020; Lowry & Lowry, 2014; Reba & Seto, 2020; Surya et al., 2021; Williams et al., 2018). Furthermore, street view images may be used to understand details of land use types (e.g., green view index, sky view factor, tree view factor, street canyons), urban mobility patterns (e.g., walking behaviors, pedestrian and road infrastructure, cycling behaviors, food environment, physical injury), and perceptions of the urban environment (e.g., environmental aesthetics, perceived safety, behaviors regarding destinations and functionality), which are low cost and highly efficient (Kang et al., 2020). These data can also be triangulated with other data sources, such as observations, questionnaires, and documentation, to address a variety of research questions (Surya et al., 2021). As none of the studies included in this review investigated the modifying or interactive role of land‐use and land‐cover change on the heat‐health relationship in the Arabian Peninsula, this research topic is crucial to study, especially given the rapid population growth and urbanization in the region.

### 4.5 Practical Actions in the Arabian Peninsula

To adequately respond to the growing health threat of extreme heat in the region, greater coordination is needed not only between the central and local governments but also between local government institutions. The overlapping of mandates and the lack of effective information exchange hinder effective multi‐hazard risk assessment and forecasting, which are indispensable for a timely response to heat waves (Bergh, 2022). Local governments can implement heat action policies that consider a multi‐level approach, from the individual‐to the community‐level, to bolster resilience across specific community requirements.

Non‐government organizations should collaborate with the authorities at the local level to undertake targeted awareness‐raising campaigns to warn susceptible groups of heat risks and to promote low‐cost interventions, such as the use of cooling scarves (Bergh, 2022). Moreover, by engaging impacted individuals in decision‐making, more effective local initiatives for climate actions may be tailored and implemented (Whitmarsh et al., 2012). An adaptation opportunity exists for building codes and standards to be revised and instituted based on local climatic and cultural condition. For example, authorities can offer subsidies for fitting awnings or other sun shelters, enhancing house temperature control (Bergh, 2022). Local governments may also provide access to heat‐related resources, such as establishing cooling stations in public spaces as well as telephone helplines in need of treatment and assistance to provide timely relief to susceptible sub‐populations (Bedi et al., 2022; Lowe et al., 2011).

Collaboration between government, academia, and non‐government agencies is needed to further evaluate and improve heat interventions based on epidemiological evidence, especially given the region's unique set of issues and continued urbanization (Hess et al., 2023; NCEH, 2020). This provides an opportunity for future planning and facilitates a drastic shift in environment‐based design in new developments and infrastructure that can render the region more resistant to the impacts of climate change. By embracing such forward‐thinking projects, the Arabian Peninsula can increase its capacity to adapt to heat exposure, safeguard vulnerable populations, and increase community resilience in the face of climate change.

### 4.6 Strengths and Limitations

To date, this is the first scoping review conducted to identify research investigating heat exposure impacts on adverse health outcomes in the Arabian Peninsula. We gathered papers published from 2010 onwards from three comprehensive databases to ensure that we captured as many studies as possible that met our criteria. Moreover, we provide recommendations based on the literature limitations, which can aid future research and improve the quality of such analyses.

There were also limitations in this review. We restricted our search to 2010–2024 to include only up‐to‐date analyses and data sets; however, there may be papers prior to 2010 that are worthwhile to assess. Additionally, we chose not to capture global papers in our primary search, given that these papers tend to have minimal or no description that specifically mentions the Arabian Peninsula. Some may nonetheless contain certain valuable information despite their generality. Thus, the final set comprises only MCC Collaborative Research Network studies; a broader set would be obtained if considering a larger portion of MENA. Furthermore, we acknowledge no strict geographical delineations define the Arabian Peninsula; in our review, we only included the southern portions of both Iraq and Jordan (Luque‐García et al., 2024; Shi et al., 2024). Given the scope of our review, we limited our evaluation to direct or chronic health impacts, hospitalizations, and mortality only; however, several studies outside of our scope investigated the impacts of heat exposure on other health outcomes including but not limited to infectious, water‐borne, vector‐borne, and food‐borne diseases. Future reviews and studies may wish to focus on these topics and their relationship to extreme heat.

## 5 Conclusion

In our scoping review on heat‐related health impacts in the Arabian Peninsula, we identified a limited number of papers studying this topic from 2010 to 2024, with much of the region having seen almost no targeted work. This stands in direct contrast to the large number of regional and global‐scale physical‐science papers highlighting the Arabian Peninsula as a hotspot of current and future extreme heat events in a warming world. While scarce in number, the existing works are critically important for identifying the significant health burden related to extreme heat in certain Arabian countries. where sufficient data are available. Our review reveals opportunities for future research questions in this domain related to geographic subregion, the temperature exposure metric, the health outcome, and analytical considerations, and we provided recommendations for future research as a result. It is imperative to assess the health burden attributed to extreme heat across sub‐populations in this region, given its intensifying heat, rapid and continued urbanization, and growing populations and economies. Further research in the Arabian Peninsula may build on this foundation to provide insight regarding the demographic and socioeconomic characteristics that underpin the heat‐health relationship and novel strategies to reduce the associated health burden.

## Conflict of Interest

The authors declare no conflicts of interest relevant to this study.

## Supporting information

Supporting Information S1

## Data Availability

This scoping review paper did not analyze any new data, and only published results in identified previous studies were used. The list of studies reviewed and information from the papers included in this scoping review is provided in Table 1 (see details in Table S1 in Supporting Information S1). Furthermore, the sensitivity analysis extraction is provided in Table S2 in Supporting Information S1. Every study included in this review is provided in the reference list.
