# Supplementary material for: Overheated and Understudied: A Scoping Review of Heat‐Related Health Impacts in the Arabian Peninsula
Source: Geohealth. 2025 Jun 30;9(7):e2024GH001277. doi: 10.1029/2024GH001277 (PMC12207249; doi:10.1029/2024GH001277)
Supplement: Supplementary file 1 — Supporting Information S1 [file GH2-9-e2024GH001277-s001.docx]

*GeoHealth*

Supporting Information for

**Overheated and Understudied: A Scoping Review of Heat-Related Health Impacts in the Arabian Peninsula**

Anais Teyton†^1-3^, Jennifer Bailey^†1-3^, Eqi Luo^4^, Rahaf Ajaj^5^, Colin Raymond^6^, Cascade Tuholske^4,7^, Tarik Benmarhnia^3,8^

^1^ Herbert Wertheim School of Public Health and Human Longevity Science, University of California, San Diego, 9500 Gilman Drive, La Jolla, CA, 92093, USA

^2^ School of Public Health, San Diego State University, 5500 Campanile Dr, San Diego, CA, 92182, USA

^3^ Scripps Institution of Oceanography, University of California, San Diego, 8885, Biological Grade, La Jolla, CA, 92037, USA

^4^ Department of Earth Sciences, Montana State University, 226 Traphagen, P.O. Box 173480. Bozeman, MT, 59717-3480, USA

^5^ College of Health Science, Abu Dhabi University, Zayed City, MZ39, Abu Dhabi, United Arab Emirates

^6^ Joint Institute for Regional Earth System Science and Engineering, University of California, Los Angeles, 4242 Young Hall, 607 Charles E Young Dr East, Los Angeles, CA 90095, USA

^7^ Geospatial Core Facility, Montana State University, Traphagen 205, P.O. Box 173480, Bozeman, MT, 59717-2220, USA

^8^ Irset Institut de Recherche en Santé, Environnement et Travail, UMR-S 1085, Inserm, University of Rennes, EHESP, Rennes, France

†Co-first author status

**Contents of this file**

Text S1

Tables S1 to S2

Text S1.

**Database Search Information**

**General Search Strategy- Exposure, Outcome, Region:**

*Search:*

(“heat wave*” OR heatwave* OR heat OR temperature* OR humidex OR “heat ind*” OR “warm spell*”)

AND

(Morbidity OR mortality OR hospital* OR “emergency department” OR “emergency service*” OR death OR emergenc* OR health)

AND

(“Arabian Peninsula” OR “Arabian Desert” OR Arabia OR “Saudi Arabia” OR Yemen OR Oman OR Kuwait OR Qatar OR Bahrain OR “United Arab Emirates” OR Jordan OR Iraq)

**PUBMED**

*Additional Information:*

Studies before 2010 removed

*Search:*

(“heat wave*”[tiab] OR heatwave*[tiab] OR heat[tiab] OR temperature*[tiab] OR humidex[tiab] OR “heat ind*”[tiab] OR “warm spell*”[tiab])

AND

(Morbidity[tiab] OR mortality[tiab] OR hospital*[tiab] OR “emergency department”[tiab] OR “emergency service*”[tiab] OR death[tiab] OR emergenc*[tiab] OR health[tiab])

AND

(“Arabian Peninsula”[tiab] OR “Arabian Desert”[tiab] OR Arabia*[tiab] OR “Saudi Arabia”[tiab] OR Yemen[tiab] OR Oman[tiab] OR Kuwait[tiab] OR Qatar[tiab] OR Bahrain[tiab] OR “United Arab Emirates”[tiab] OR Jordan[tiab] OR Iraq[tiab])

NOT(Autobiography[Publication Type])

NOT(Bibliography[Publication Type])

NOT(Biography[Publication Type])

NOT(Case Reports[Publication Type])

NOT(Comment[Publication Type])

NOT(Review[Publication Type])

NOT(Editorial[Publication Type])

NOT(News[Publication Type])

**WEB OF SCIENCE**

*Additional Information:*

DT=(Article)

AND restricted to 2010 and on

*Search:*

TI=((“heat wave*” OR “heatwave*” OR heat OR temperature* OR humidex OR “heat ind*” OR “warm spell*”)

AND

(Morbidity OR mortality OR hospital* OR “emergency department” OR “emergency service*” OR death OR emergenc* OR health)

AND

(“Arabian Peninsula” OR “Arabian Desert” OR Arabia OR “Saudi Arabia” OR Yemen OR Oman OR Kuwait OR Qatar OR Bahrain OR “United Arab Emirates” OR Jordan OR Iraq))

OR

AB=((“heat wave*” OR heatwave* OR heat OR temperature* OR humidex OR “heat ind*” OR “warm spell*”)

AND

(Morbidity OR mortality OR hospital* OR “emergency department” OR “emergency service*” OR death OR emergenc* OR health)

AND

(“Arabian Peninsula” OR “Arabian Desert” OR Arabia OR “Saudi Arabia” OR Yemen OR Oman OR Kuwait OR Qatar OR Bahrain OR “United Arab Emirates” OR Jordan OR Iraq))

**SCOPUS**

*Additional information:*

N/A

*Search:*

TITLE-ABS("heat wave*" OR heatwave* OR heat OR temperature* OR humidex OR "heat ind*" OR "warm spell*")

AND TITLE-ABS(Morbidity OR mortality OR hospital* OR "emergency department" OR "emergency service*" OR death OR emergenc* OR health)

AND TITLE-ABS("Arabian Peninsula" OR "Arabian Desert" OR Arabia OR "Saudi Arabia" OR Yemen OR Oman OR Kuwait OR Qatar OR Bahrain OR "United Arab Emirates" OR Jordan OR Iraq)

AND PUBYEAR > 2009

AND PUBYEAR < 2025

AND (

LIMIT-TO ( DOCTYPE,"ar" )

)

AND (

LIMIT-TO ( LANGUAGE,"English" )

Table S1. Extracted data from selected studies in primary search (n=9), including information about the study methodology (e.g., study location, study design, time period assessed, type of analysis, statistical design, sample size, and sampling strategy), temperature exposure (e.g., type, measure, temporal dimension, and geographical scale), the health outcome (measurement, temporal dimension, and geographical scale), and analytical considerations (effect estimate, confounders, effect modification and landscape and urban form).

| **Paper Citation** | **Country** | **Time Period** | **Study Design** | **Type of Analysis & Statistical Design** | **Sample Size** | **Sampling Strategy** | **Temperature (T) Exposure** | **T Exposure Measurement** | **Duration of T Exposure** | **Geographical Scale of T Exposure** | **Health Outcome Measurement** | **Temporal Dimension of Health Outcome** | **Geographical Dimension of Health Outcome** | **Effect Estimate** | **Confounders** | **Effect Modification** | **Effect Modification and/or Interaction by Landscape & Urban Form** |
| --- | --- | --- | --- | --- | --- | --- | --- | --- | --- | --- | --- | --- | --- | --- | --- | --- | --- |
| Alahmad, B., Shakarchi, A., Alseaidan, M., & Fox, M. (2019).  The effects of temperature on short-term mortality risk in Kuwait: a time-series analysis | Kuwait | 2010-2016 | Longitudinal (Time-series design) | Quantitative  Distributed lag non-linear models (DLNM) to capture delayed effects of temperature on mortality over a span of 30 days. | Entire population included  33,574 all-cause non-accidental deaths | All events included (all-cause non-accidental deaths) | Outdoor average temperature | Meteorological data (n=15 meteorological stations) | Daily | Multi-city | All-cause non-accidental mortality | Daily | Ecological | Risk Ratios (all-cause non-accidental morality): **42.7°C (99th percentile) vs. optimum T (66th percentile, 34.7°C):** RR = 1.63; 95% CI: 1.09 to 2.46 **41.6 °C (97.5th percentile) vs. optimum T:** RR = 1.41; 95% CI: 1.02 to 1.93 | Ozone, PM10, RH, year, & day of the week. Temporally stable confounders adjusted for by design. | None | None |
| Pradhan, B., Kjellstrom, T., Atar, D., Sharma, P., Kayastha, B., Bhandari, G., & Pradhan, P. K. (2019).  Heat stress impacts on cardiac mortality in Nepali migrant workers in Qatar | Qatar | 2009-2017 | Longitudinal (Time-series design) | Mixed Methods  Descriptive statistics (Pearson correlation & Student’s t test), simple linear regression, & qualitative interviews. | All Nepali migrant workers  Over 1,300 Nepali migrant workers (NMW) died during study period | All events included (all deaths of Nepali migrant workers) | Outdoor maximum wet bulb globe temperature (WBGT; "in-shade" as indoors or full shade, without solar or other heat radiation) | Meteorological data (n=1 meteorological station) | Monthly | Single city | Cardiovascular-specific mortality | Monthly | Ecological | Beta coefficients (cardiovascular deaths per 100,000 NMWs): **1-Month WBGT (in-shade):** β = 5.5**;** 95% CI not explicitly stated **3-Month WBGT (in-shade):** β = 5.7; 95% CI not explicitly stated | None | None | None |
| Alahmad, B., Khraishah, H., Shakarchi, A. F., Albaghdadi, M., Rajagopalan, S., Koutrakis, P., & Jaffer, F. A. (2020).  Cardiovascular mortality & exposure to heat in an inherently hot region: implications for climate change | Kuwait | 2010-2016 | Longitudinal (Time-series design) | Quantitative  DLNMs used to capture delayed effects of temperature on mortality over a span of 30 days. | Entire population included  15,609 total cardiovascular-related deaths | All events included (all cardiovascular-related deaths) | Outdoor average temperature | Meteorological data (n=15 meteorological stations) | Daily | Multi-city | Cardiovascular-specific mortality | Daily | Ecological | Risk Ratios (cardiovascular mortality): **42.7°C (99th percentile) vs. MMT (34.7°C):** RR = 3.09; 95% CI: 1.72 to 5.55 | Ozone, PM10, RH, year, seasonality, & day of the week. Temporally stable confounders adjusted for by design. | **Age:** Aged 15–64 years: RR = 3.84; 95% CI: 1.57 to 7.70 Aged above 65: RR = 2.29; 95% CI: 0.96 to 5.48   **Sex:** Males: RR = 3.53; 95% CI: 1.74–7.16 Females: RR = 2.36; 95% CI: 0.83 to 6.66 | None |
| Alahmad, B., Shakarchi, A. F., Khraishah, H., Alseaidan, M., Gasana, J., Al-Hemoud, A., ... & Fox, M. A. (2020).  Extreme temperatures & mortality in Kuwait: who is vulnerable? | Kuwait | 2010-2016 | Longitudinal (Time-series design) | Quantitative  DLNMs used to capture delayed effects of temperature on mortality over a span of 30 days. | Entire population included  33,472 all-cause non-accidental deaths | All events included (all-cause non-accidental & cardiovascular-specific deaths) | Outdoor average temperature | Meteorological data (n=15 meteorological stations) | Daily | Multi-city | All-cause non-accidental & cardiovascular mortality | Daily | Ecological | Risk Ratios (non-accidental mortality): **42.7°C (99th percentile) vs. MMT (66th percentile, 34.7°C):** RR = 1.63; 95% CI: 1.09 to 2.46  Risk Ratios (cardiovascular-specific mortality):  **42.7°C (99th percentile) vs. MMT (66th percentile, 34.7°C):**  RR = 3.09; 95% CI: 1.72 to 5.55 | Ozone, PM10, RH, year, & day of the week. Temporally stable confounders adjusted for by design. | **Total non-accidental mortality (sex, age, & nationality):** Males: RR = 2.08; 95% CI: 1.23 to 3.52  Females: RR = 1.14; 95% CI: 0.60 to 2.18 0-14 years old: RR = 0.29; 95% CI: 0.09 to 0.97 15-64 years old: RR = 2.28; 95% CI: 1.24 to 4.20 65+ years old: RR = 1.86; 95% CI: 1.02 to 3.39 Kuwaiti: RR = 1.38; 95% CI: 0.80 to 2.41 Non-Kuwaiti: RR = 1.96; 95% CI: 1.10 to 3.52  **Cardiovascular mortality (sex, age, & nationality):**  Males: RR = 3.53; 95% CI: 1.74 to 7.16 Females: RR = 2.36; 95% CI: 0.83 to 6.66 0-14 years old: omitted due to wide SEs 15-64 years old: RR = 3.84; 95% CI: 1.57 to 7.70 65+ years old: RR = 2.29; 95% CI: 0.96 to 5.48 Kuwaiti: RR = 2.98; 95% CI: 1.23 to 7.20 to  Non-Kuwaiti: RR = 3.15; 95% CI: 1.44 to 6.91 | None |
| Al-Bouwarthan, M., Quinn, M. M., Kriebel, D., & Wegman, D. H. (2020).  A field evaluation of construction workers’ activity, hydration status, & heat strain in the extreme summer heat of Saudi Arabia | Saudi Arabia | 2016  (June to September) | Prospective cohort study | Quantitative  Linear mixed models with a stepwise method to select which exposures of heat/workload are included in each model. | 23 construction workers | Non-random sampling | Indoor & outdoor average WBGT   Heat Stress Exceedence (HSE) | Portable sensor-based | Daily | Individual | Heart Rate Reserve (HRR, as a measure of cardiovascular strain, %) | Daily | Individual | Beta coefficients (HRR): **WBGT:** β = 0.78; 95 CI%: 0.45 to 1.12 **HSE:** β = 0.70; 95 CI%: 0.36 to 1.04 | None | None | None |
| Alghamdi, A. S., Alqadi, A., Alghamdi, F., Jenkins, R. O., & Haris, P. I. (2021).  Higher ambient temperature is associated with worsening of HbA1c levels in a Saudi population | Saudi Arabia | 15 November 2012 - 9 September 2018 | Retrospecitve cohort | Quantitative  Descriptive statistics (including Welch's ANOVA with post hoc analysis using Games-Howell tests, unparied t-tests, Pearson Correlation, & chi-square test), multiple linear regression models, & a subsequent logistic regression model. | 168,614 adult Saudi patients at the Security Forces Hospital in Riyadh who had HbA1c analyzed | All events included (all patients who had HbA1c analyzed) | Outdoor maximum temperature  Categorical outdoor maximum T (high (> 35.8°C), moderate (26.1°C-35.8°C), & low (< 26.1°C)) | Meteorological data (number of meteorological stations not stated) | Weekly | Single city | HbA1c values (%) divided into 2 categories: HbA1c < 7% (good glycemic control) & HbA1c ≥ 7% (poor glycemic control) | Single occurrence | Individual | Odds Ratios (HbA1c ≥ 7% (poor glycemic control)): **High T (> 35.8°C) vs. low T (< 26.1°C):**  OR = 1.134; 95% CI: 1.107 to 1.162 **Moderate T (26.1°C-35.8°C) vs. low T (< 26.1°C):**  OR = 1.034; 95% CI: 1.007 to 1.062 | Age & gender | None | None |
| Alahmad, B., Vicedo-Cabrera, A. M., Chen, K., Garshick, E., Bernstein, A. S., Schwartz, J., & Koutrakis, P. (2022).  Climate change & health in Kuwait: temperature & mortality projections under different climatic scenarios | Kuwait | 2000-2016 | Longitudinal (Time-series design & projection) | Quantitative  DLNMs used to estimate the baseline temperature-mortality relationship (30 day temperature lag) using quasi-Poisson regression. Quantified the temperature-related mortality burdens in the mid- (2050–2059) & end-century (2090–2099) decades under moderate & extreme climate change scenarios. | Entire population included  73,748 all-cause non-accidental deaths & 35,285 cardiovascular deaths | All events included (all-cause non-accidental & cardiovascular deaths) | Outdoor average temperature | Meteorological data (n=1 meteorological station) | Daily | Single city | All-cause non-accidental & cardiovascular mortality | Daily | Ecological | Non-linear U-shaped temperature-mortality relationships identified, but effect estimates not explicitly stated. | RH, year, seasonality, & day of the week. Temporally stable confounders adjusted for by design. | Age (0-65 & 65+), gender (men & women), & nationality (Kuwaitis & Non-Kuwaitis). Those aged above 65 years & non-Kuwaitis found to be at increased risk.  Non-linear, predominantly U-shaped temperature-mortality curves identified, but effect estimates not explicitly stated. | None |
| Alahmad, B., Al-Hemoud, A., Al-Bouwarthan, M., Khraishah, H., Kamel, M., Akrouf, Q., ... & Koutrakis, P. (2023).  Extreme heat & work injuries in Kuwait’s hot summers | Kuwait | 2015-2019 (1 June to 31 August) | Longitudinal (Time-series design) | Quantitative  Generalised additive models with a quasi-Poisson distribution & a 3-,5-, & 7-day moving average of daily temperature. | All private sector workers in Kuwait  3,710 occupational injuries | All events included (occupational injuries reported by the private sector) | Outdoor average temperature | Meteorological data (n=1 meteorological station) | Daily | Single city | Occupational injuries | Daily | Ecological | Risk Ratios (Occupational injuries): **39.4°C vs. 37.0°C (10th percentile T):**  RR = 1.44; 95% CI: 1.34 to 1.53 **40°C vs. 37.0°C:** RR = 1.48; 95% CI: 1.39 to 1.59 **41°C vs. 37.0°C:** RR = 1.44; 95% CI: 1.27 to 1.63 **42°C (90th percentile T) vs. 37.0°C:**  RR = 1.21; 95% CI: 0.93 to 1.57 | RH, year, seasonality, & day of the week. Temporally stable confounders adjusted for by design. | None | None |
| Yezli, S., Khan, A. H., Yassin, Y. M., Khan, A. A., Alotaibi, B. M., & Bouchama, A. (2023).  Human tolerance to extreme heat: evidence from a desert climate population | Saudi Arabia | 1 February 2006 to 1 October 2015 | Longitudinal (Time-series design) | Quantitative  DLNM used to estimate the mortality-temperature association over 25 lag days. | Population of Mecca, Saudi Arabia  37,178 non-accidental deaths | All events included (all non-accidental deaths) | Outdoor average temperature | Meteorological data (n=1 meteorological station) | Daily | Single city | All-cause non-accidental mortality | Daily | Ecological | Non-linear U-shaped temperature-mortality relationship identified, but effect estimates not explicitly stated.   **38°C (97.5th percentile T) vs. MMT (48th percentile, 31.8°C):** Attributable Mortality = 5.6%; 95% CI: -3.8 to 13.2 | Year, Hajj-Season, Ramadan-Season, month, day of week, & Hajj-Ritual-Day. Temporally stable confounders adjusted for by design. | None | None |
| Alahmad, B., Ali, H., Alwadi, Y., Al-Hemoud A, Koutrakis, P., & Al-Mulla, F. (2024).  Combined impact of heat & dust on diabetes hospitalization in Kuwait | Kuwait | 2010-2019 (June - August) | Longitudinal (Case-crossover design) | Quantitative  A case-crossover design was used, where distributed lag non-linear models assessed the relationship between temperature (7 day lag) and diabetes hospitalization. Generalized additive model to assess interaction between 7-day moving average of temperature & dust on diabetes hospitalizations. | All individuals with unplanned hospital admissions for diabetes mellitus  8,960 unplanned hospitalizations | All events included (all unplanned hospital admissions for diabetes mellitus from public hospitals (n=14)) | Outdoor average temperature | Meteorological data (n=1 meteorological station) | Daily | Single city (extrapolated to the country level) | Diabetes mellitus hospitalization | Daily | Ecological | Risk ratios (hospitalizations for diabetes): **34°C vs. 33°C (lowest summer T, rounded to the whole number):**  RR = 1.11; 95% CI: 1.01 to 1.23 **38°C vs. 33°C:** RR = 1.58; 95% CI: 1.03 to 2.42 **44°C vs. 33°C:** RR = 1.52; 95% CI: 0.74 to 3.13  Identified a non-linear relationship between heat & diabetic hospitalizations during summer months (see RR estimates above)  Excess diabetic admissions attributed to hot days above 33°C each year = 282; 95% CI: -14 to 473 | Year, month, & day of the week. Temporally stable confounders adjusted for by design. | **34°C vs. 33°C (lowest summer T) (sex & age):** Male: RR = 1.13; 95% CI: 0.99 to 1.29 Female: RR = 1.09; 95% CI: 0.95 to 1.26 Elderly (65+): RR = 1.10; 95% CI: 0.90 to 1.35  **38°C vs. 33°C (sex & age):** Male: RR = 1.62; 95% CI: 0.91 to 2.88  Female: RR = 1.50; 95% CI: 0.81 to 2.78 Elderly (65+): RR = 1.58; 95% CI: 0.65 to 3.84  **44°C vs. 33°C (sex & age):** Male: RR = 1.40; 95% CI: 0.52 to 3.78  Female: RR = 1.53; 95% CI: 0.55 to 4.26  Elderly (65+): RR = 2.39; 95% CI: 0.58 to 9.86 | None |
| Alwadi, Y., Al-Hemoud, A., Khraishah, H., Al-Mulla, F., Koutrakis, P., Ali, H., & Alahmad, B. (2024).  Impact of Extreme Heat on Cardiovascular Health in Kuwait: Present & Future Projections | Kuwait | 2010-2019 | Longitudinal (Time-series design) | Quantitative  Distributed lag non-linear models to assess relationship between temperature (21 day lag) and cardiovascular disease hospitalizations. Temperature projections assessed for every decade through 2099 for moderate & extreme climate change scenarios. | Entire population included  263,182 cardiovascular hospitalizations | All events included (cardiovascular-related hospital admissions) | Outdoor average temperature | Meteorological data (n=1 meteorological station) | Daily | Single city (extrapolated to the country level) | Cardiovascular-specific hospital admissions for all cardiovascular diseases, ischemic heart disease, & stroke | Daily | Ecological | Risk Ratios (all cardiovascular hospitalizations): **41°C vs. MMT (20.7°C):** RR = 1.292; 95% CI: 1.051 to 1.589 **42°C vs. MMT :** RR = 1.309; 95% CI: 1.036 to 1.654 **43°C vs. MMT:** RR = 1.326; 95% CI: 1.006 to 1.747 Attributable admissions for all hot days above the MMT = 20,569; 95% CI: 3,128 to 35,757  Risk Ratios (ischemic heart disease hospitalizations): **41°C vs. MMT (18.4 °C):** RR = 1.378; 95% CI: 1.046 to 1.815 **42°C vs. MMT:** RR = 1.433; 95% CI: 1.052 to 1.951 **43°C vs. MMT:** RR = 1.495; 95% CI: 1.042 to 2.144 Attributable admissions for all hot days above the MMT = 13,741; 95% CI: -1,941 to 26,169  Risk Ratios (stroke hospitalizations): **41°C vs. MMT (21.7 °C):** RR = 1.212; 95% CI: 0.780 to 1.882 **42°C vs. MMT:** RR = 1.219; 95% CI: 0.756 to 1.965 **43°C vs. MMT:** RR = 1.225; 95% CI: 0.730 to 2.056 Attributable admissions for all hot days above the MMT = 2,379; 95% CI: -2,533 to 6,287  Non-linear, somewhat U-shaped temperature-hospitalization relationships identified. | RH, seasonality, time trends (not explicitly stated), & day of the week. Temporally stable confounders adjusted for by design. | None | None |
| Zhao, Q., Li, S., Ye, T., Wu, Y., Gasparrini, A., Tong, S., ... & MCC Collaborative Research Network. (2024).   Global, regional, & national burden of heatwave-related mortality from 1990 to 2019: A three-stage modelling study. | Kuwait, Oman, Saudi Arabia, United Arab Emirates, Yemen | Kuwait: 2000-2016 (warm season)  Other countries: not explicitly stated | Longitudinal (Time-series design) | Quantitative  Three-stage modelling approach to estimate heatwave-mortality relationship in each location using a quasi-Poisson regression with constrained distributed lag model (lag up to 10 days), multivariate meta-regression for location-specific associations and predictors, grid-specific associations estimated using the meta-regression results and 5 meta-predictors per decade. | Entire population included  Deaths: N/A | All events included (all-cause &/or non-external deaths, not explicitly stated per country) | Outdoor average temperature   Heat wave defined as daily mean temperature ≥95th percentiles of year-round temperature range with duration ≥2 days | Global gridded dataset: from Climate Prediction Center Global Unified Temperature Data (0.5°×0.5° resolution) | Daily | Single city (extrapolated to the country level) | All-cause &/or non-external mortality (not specified at the country level) | Daily | Ecological | Effect estimates not explicity provided at the country-level. | Decade, year, seasonality, & day of the week. Temporally stable confounders adjusted for by design. | None | None |

Notes: T = temperature; DLNM = distributed lag non-linear model; RR = risk ratio; PM10 = particulate matter ≤ 10 micrometers; RH = relative humidity; NMW = Nepali migrant workers; WBGT = wet bulb globe temperature; MMT = minimum mortality temperature; SE = standard error; HSE = heat stress exceedence; HRR = Heart Rate Reserve; β = beta estimates; OR = odds ratio

Table S2. Extracted data from selected studies from the sensitivity analysis (n=9), including information about the study methodology (e.g., study location, study design, time period assessed, type of analysis, statistical design, sample size, and sampling strategy), temperature exposure (e.g., type, measure, temporal dimension, and geographical scale), the health outcome (measurement, temporal dimension, and geographical scale), and analytical considerations (effect estimate, confounders, effect modification and landscape and urban form).

| **Paper Citation** | **Country** | **Time Period** | **Study Design** | **Type of Analysis & Statistical Design** | **Sample Size** | **Sampling Strategy** | **Temperature (T) Exposure** | **T Exposure Measurement** | **Duration of T Exposure** | **Geographical Scale of T Exposure** | **Health Outcome Measurement** | **Temporal Dimension of Health Outcome** | **Geographical Dimension of Health Outcome** | **Effect Estimate** | **Confounders** | **Effect Modification** | **Effect Modification and/or Interaction by Landscape & Urban Form** |
| --- | --- | --- | --- | --- | --- | --- | --- | --- | --- | --- | --- | --- | --- | --- | --- | --- | --- |
| Tobías, A., Hashizume, M., Honda, Y., Sera, F., Ng, C. F. S., Kim, Y., ... & Gasparrini, A. (2021).   Geographical variations of the minimum mortality temperature at a global scale: a multicountry study | Kuwait | 2000-2016 | Longitudinal (Time-series design) | Quantitative  A two-stage design: 1) estimated temperature-mortality associations to derive the minimum mortality temperature (MMT) for each location using Poisson regression with distributed lag nonlinear models (DLNMs), with a 21 day lag; & 2) assessed MMT variation by climatic zone using a mixed-effects meta-analysis & explored the association with climatic & socioeconomic indicators. | Entire population included  Deaths: N/A | All events included (all non-external deaths) | Outdoor average temperature | Meteorological data (number of meteorological stations not stated) | Daily | Single city (extrapolated to the country level) | Non-external mortality | Daily | Ecological | A non-linear U-shaped temperature-mortality relationship identified, but effect estimates not explicitly stated. | Year, seasonality, & day of the week. Temporally stable confounders adjusted for by design. | None | None |
| Vicedo-Cabrera, A. M., Scovronick, N., Sera, F., Royé, D., Schneider, R., Tobias, A., ... & Gasparrini, A. (2021).   The burden of heat-related mortality attributable to recent human-induced climate change | Kuwait | 2000-2016 (June-September) | Longitudinal (Time-series design) | Quantitative  A two-stage design: 1) quasi-Poisson regression with DLNMs to estimate effects of location-specific temperature on mortality over 10 days; & 2) a multivariate metaregression model to pool coefficients. A factual scenario was calculated consisting of simulations of all climate forcings & a counterfactual scenario where climate simulations are driven by natural forcings only. | Entire population included  22,347 total non-external deaths | All events included (all non-external deaths) | Outdoor average temperature | Meteorological data (number of meteorological stations not stated)  Global gridded dataset: from the Detection & Attribution Model Intercomparison Project (component of CMIP6, resolution not explicitly stated) | Daily | Single city (extrapolated to the country level) | Non-external mortality | Daily | Ecological | A non-linear, somewhat U-shaped temperature-mortality relationship identified, but effect estimates not explicitly stated. | Decade, year, seasonality, & day of the week. Temporally stable confounders adjusted for by design. | None | None |
| Zhao, Q., Guo, Y., Ye, T., Gasparrini, A., Tong, S., Overcenco, A., ... & Li, S. (2021).   Global, regional, & national burden of mortality associated with non-optimal ambient temperatures from 2000 to 2019: a three-stage modelling study | Kuwait | 2000-2016 | Longitudinal (Time-series design) | Quantitative  A three-stage design: 1) temperature-mortality association fitted with a quasi-Poisson regression with a DLNM (21 day lag) for each location; 2) multivariate meta-regression model used between location-specific estimates & meta-predictors; & 3) grid-specific temperature-mortality relationship predicted using the fitted meta-regression & the grid-specific meta-predictors. | Entire population included  73,748 total deaths | All events included (all-cause &/or non-external deaths, not explicitly stated per country) | Outdoor average temperature | Global gridded dataset: from the Climate Prediction Center (0.5°×0.5° resolution) | Daily | Single city (extrapolated to the country level) | Non-external mortality | Daily | Ecological | Effect estimates not explicitly provided. | Year, seasonality, & day of the week. Temporally stable confounders adjusted for by design. | None | None |
| Mistry, M. N., Schneider, R., Masselot, P., Royé, D., Armstrong, B., Kyselý, J., ... & Gasparrini, A. (2022).   Comparison of weather station & climate reanalysis data for modelling temperature-related mortality | Kuwait | 2000-2016 | Longitudinal (Time-series design) | Quantitative  A two-stage design: 1) location-specific temperature-mortality associations were calculated using quasi-Poisson with DLNMs (21 day lag) & compared using ground weather stations observations & reanalysis data; & 2) the location-specific coefficients were then pooled in a multivariate multilevel meta-regression model. | Entire population included  73,748 non-external deaths | All events included (all non-external deaths) | Outdoor average temperature | Meteorological data (nearest weather station used)  Global gridded datasets: from Copernicus ERA5-Land climate reanalysis data (0.09° resolution) & ERA5 (0.25° resolution) | Daily | Single city (extrapolated to the country level) | Non-external mortality | Daily | Ecological | Risk ratios (non-external mortality, 99th percentile T*): **ERA5-Land:** RR = 1.08; 95% CI: 0.92 to 1.26 **ERA5:** RR = 1.07; 95% CI: 0.93 to 1.24 **Station:** RR = 1.12; 95% CI: 0.92 to 1.36 * 99th percentile T not explicitly stated   A non-linear, somewhat U-shaped temperature-mortality relationship identified. | Year, seasonality, & day of the week. Temporally stable confounders adjusted for by design. | None | None |
| Alahmad, B., Khraishah, H., Royé, D., Vicedo-Cabrera, A. M., Guo, Y., Papatheodorou, S. I., ... & Koutrakis, P. (2023).   Associations between extreme temperatures & cardiovascular cause-specific mortality: results from 27 countries | Kuwait | 2000-2016 | Longitudinal (Case-crossover design) | Quantitative  A two-stage design: (1) estimated the association between extreme temperatures & cardiovascular-specific deaths using quasi-Poisson regression with DLNMs (14 day lag); & (2) pooled all risk estimates from cities for an overall estimate. | All cases included  35,285 all cardiovascular disease (CVD) deaths; 17,251 ischemic heart disease deaths; 6,615 stroke deaths; 4,620 heart failure deaths; & 234 arrhythmia deaths | All events included (all cardiovascular-specific deaths) | Outdoor average temperature | Meteorological data (number of meteorological stations not stated) | Daily | Single city (extrapolated to the country level) | Cardiovascular-specific mortality for all CVD, ischemic heart disease, stroke, heart failure, & arrhythmia | Daily | Ecological | Risk ratios (cardiovascular-specific mortality, 99th percentile T vs. MMT*): **All CVD:** RR = 1.17; 95% CI: 1.08 to 1.27 **Ischemic Heart Disease:** RR = 1.14; 95% CI: 1.04 to 1.24 **Stroke:** RR = 1.07; 95% CI: 0.96 to 1.18 **Heart Failure:** RR = 1.05; 95% CI: 0.84 to 1.32 **Arrythmia:** RR = 0.94; 95% CI: 0.73 to 1.21 *99th percentile T = 42.08°C & MMT = 37.33°C (for all-cause cardiovascular deaths)  Excess death (per 1000 deaths) attributable to a range of extreme hot temperatures (97.5th percentile T & above) & attributable to all hot temperatures (above the MMT) also reported. | Year, month, & day of the week. Temporally stable confounders adjusted for by design. | None | None |
| Lo, Y. E., Mitchell, D. M., Buzan, J. R., Zscheischler, J., Schneider, R., Mistry, M. N., ... & Vicedo‐Cabrera, A. M. (2023).   Optimal heat stress metric for modelling heat‐related mortality varies from country to country | Kuwait | 2000-2016 (June - September) | Longitudinal (Time-series design) | Quantitative  A two-stage design: 1) quasi-Poisson regression with DLNMs (10 day lag) used to derive exposure-response associations between 8 heat exposure metrics & all-cause mortality over the warm season; & 2) pooled derived location-specific exposure-response curves in a multivariate meta-regression model used to sum daily heat-related deaths across all days in the warm season. | Entire population included  22,347 non-external deaths | All events included (all non-external deaths) | Dry-bulb average temperature (T mean)  Heat stress metrics: mean wet bulb temperature (Tw), apparent temperature (AT), discomfort index (DI), & cooling efficiency temperatures (Swmp at 20%, 40%, 60%, & 80% efficiencies) | Global gridded dataset: from Copernicus ERA5 climate reanalysis (resolution not explicitly stated) | Daily | Single city (extrapolated to the country level) | Non-external mortality | Daily | Ecological | Attributable fractions provided: **Attributable fraction for the best fit metric (swmp20):** 3.97; 95% CI: 1.23 to 6.51 **Attributable fraction for T mean:** 4.86; 95% CI: 1.95 to 7.39  Note: Explicit values for attributable fractions for all heat metrics not provided except for the best fit metric & mean temperature. The attributable fraction is represented by the area under the exposure‐response curves between the heat stress value of minimum mortality risk (MMT) & extreme heat stress, which are not explicitly provided for Kuwait.   Risk ratio curves for dry bulb temperature & heat stress indicators provided (linear relationships identified for Tmean & Swmp20, nonlinear relationships identified for Tw, AT, DI, & Swmp80 with increasing heat stress), explicit RR estimates not provided. | Decade, year, day of the year (seasonality), & day of the week. Temporally stable confounders adjusted for by design. | None | None |
| Rai, M., Stafoggia, M., De'Donato, F., Scortichini, M., Zafeiratou, S., Fernandez, L. V., ... & Breitner, S. (2023).   Heat-related cardiorespiratory mortality: effect modification by air pollution across 482 cities from 24 countries | Kuwait | 2000-2016 (selected the 6 consecutive hottest months, not explicitly stated) | Longitudinal (Time-series design) | Quantitative  Used an over-dispersed Poisson regression model to estimate the heat impact on cardio-respiratory mortality (2 day lag). After, a two-stage design was used: 1) Interaction between heat and air pollutants assessed using a non-parametric response-surface model (2 day lag); & 2) pooled the location-specific effect estimates using a multilevel meta-analytical model that accounts for variations in risk across two nested groups (cities & countries) to obtain country-wide & overall pooled estimates. | Entire population included  35,285 cardiovascular deaths & 5,715 respiratory deaths | All events included (all cardio-respiratory deaths) | Outdoor average temperature | Meteorological data (number of meteorological stations not stated) | Daily | Single city (extrapolated to the country level) | Cardiovascular-specific & respiratory-specific mortality | Daily | Ecological | Percent change (heat-related mortality when the 2-day mean summer T rises from 75th (36.5°C) to 99th (42.1°C) percentile of T): **CVD Mortality:** 13.25%; 95% CI: 3.20 to 23.30 **RD Mortality:** 26.38%; 95% CI: 5.92 to 46.83 | Sub-seasonal trends & day of the week. Temporally stable confounders adjusted for by design. | Percent change in heat-related CVD or RD mortality per an increase in the 2-day mean T from the 75th (36.5°C) to the 99th (42.1°C) percentile of T during **low, medium, & high air pollution days** (days with 2-day mean air pollutant concentration as 5th (60.7 µg/m³; 45.6 µg/m³), 50th (128.3 µg/m³; 87.9 µg/m³), & the 95th (539.5 µg/m³; 145.0 µg/m³) percentile of air pollution for PM10 (24-hour mean) & O3 (daily maximum 8-hour running average)).  **CVD mortality:** Low PM10: -0.83%; 95% CI: -6.42 to 4.76 Medium PM10: -0.74%; 95% CI: -5.78 to 4.30 High PM10: 1.88%; 95% CI: -6.40 to 10.16 Low O3: -0.67%; 95% CI: -7.12 to 5.79 Medium O3: -0.21%; 95% CI: -5.25 to 4.82 High O3: 0.32%; 95% CI: -6.55 to 7.18  **RD mortality:** Low PM10: -7.49%; 95% CI: - 23.95 to 8.97 Medium PM10: -6.71%; 95% CI: -21.54 to 8.13 High PM10: -3.34%; 95% CI: -29.61 to 22.93 Low O3: -15.77%; 95% CI: -27.70 to -3.84 Medium O3: -7.09%; 95% CI: -17.37 to 3.18 High O3: 4.16%; 95% CI: -10.80 to 19.11 | None |
| Alahmad, B., Khraishah, H., Kamineni, M., Royé, D., Papatheodorou, S. I., Vicedo-Cabrera, A. M., Guo, Y., Lavigne, E., Armstrong, B., & Sera, F. (2024).   Extreme Temperatures & Stroke Mortality: Evidence From a Multi-Country Analysis | Kuwait | 2000-2016 | Longitudinal (Case-crossover design) | Quantitative  A two-stage design: 1) Conditional quasi-Poisson regression model with DLNMs (14 day lag) used to identify the associations between temperature & stroke-specific mortality for each city; & 2) the cumulative risk across each city was pooled using a mixed effect meta-analysis. | All cases included  4,432 ischemic stroke deaths & 1,937 hemorrhagic stroke deaths | All events included (hemorrhagic & ischemic stroke mortality) | Outdoor average temperature | Meteorological data (number of meteorological stations not stated) | Daily | Single city (extrapolated to the country level) | Ischemic & hemorrhagic stroke mortality | Daily | Ecological | Risk ratio of stroke deaths (99th percentile* vs MMT): **Ischemic stroke (MMT: 37.9°C):** RR = 1.00; 95% CI: 0.83 to 1.22 **Hemorrhagic stroke (MMT: 39.5°C):** RR = 1.16; 95% CI: 0.97 to 1.38  * 99th percentile temperatures not stated  Excess deaths: Attributable to the hottest 2.5% of temperatures:  **Ischemic stroke deaths (per 1,000)** = -0.80; 95% CI: -5.13 to 2.13 **Hemorrhagic stroke deaths (per 1,000)** = 3.74; 95% CI: -1.91 to 7.88  Attributable to all non-optimal hot temperatures (above the MMT):  **Ischemic stroke deaths (per 1,000)** = -0.82; 95% CI: -5.16 to 2.18 **Hemorrhagic stroke deaths (per 1,000)** = 4.55; 95% CI: -2.50 to 9.89   They state that both types of strokes exhibited a U-shaped relationship with a higher risk of death at both high & low temperatures, however, the country-specific exposure-response curve was not included. | Year, month, & day of the week. Temporally stable confounders adjusted for by design. | None | None |
| Chen, K., de Schrijver, E., Sivaraj, S., Sera, F., Scovronick, N., Jiang, L., ..., & Vicedo-Cabrera, A. (2024).  Impact of population aging on future temperature-related mortality at different global warming levels | Kuwait | 2000-2014 | Longitudinal (Time-series design) | Quantitative  A two-stage design: 1) time series analysis was used to assess the location-age-specific temperature-mortality associations using quasi-Poisson regression with DLNMs (21 day lag). 2) A multivariate random meta-regression was used to pool the city-specific effects. Heat-related & cold-related excess deaths were predicted for 20-year future periods using projections & baseline daily mortality projections. | Entire population included  63,184 non-external deaths | All events included (non-external deaths) | Outdoor average temperature | Meteorological data (number of meteorological stations not stated) | Daily | Single city (Kuwait, Kuwait) | Non-external mortality | Daily | Ecological | A non-linear, somewhat U-shaped temperature-mortality relationship identified, but effect estimates not explicitly stated. | Year, seasonality, & day of the week. Temporally stable confounders adjusted for by design. | Age (<65 years, 65−74 years, >74 years) assessed, similar to the total population. Individuals >74 years had higher risk ratios, followed by 65-74, & <65 years.  Non-linear, somewhat U-shaped temperature-mortality curves identified, but effect estimates not explicitly stated. | None |
| Hundessa, S., Huang, W., Zhao, Q., Wu, Y., Wen, B., Alahmad, B., ..., & MCC Collaborators (2024).  Global & Regional Cardiovascular Mortality Attributable to Nonoptimal Temperatures Over Time | Kuwait | 2000-2016 | Longitudinal (Time-series design) | Quantitative  A three-stage design: 1) the relationship between daily average temperature & cardiovascular mortality was estimated for each location of interest using a quai-Poisson regression with a DLNM (21 day lag); 2) a multivariate meta-regression model identifed heterogeneity in the temperature-mortality relationship across all locations of interest using five meta-predictors; & 3) the temperature-cardiovascular mortality relationship was estimated for each grid at the daily level to calculate the number of daily excess deaths. | Entire population included  35,285 cardiovascular deaths | All events included (cardiovascular-specific deaths) | Outdoor average temperature | Global gridded dataset: from Climate Prediction Center Global Unified Temperature Data (55x55km^2^ resolution) | Daily | Single city (extrapolated to the country level) | Cardiovascular mortality | Daily | Ecological | A non-linear, somewhat U-shaped temperature-mortality relationship identified, but effect estimates not explicitly stated. | Year, seasonality, & day of the week. Temporally stable confounders adjusted for by design. | None | None |
| Orlov, A., De Hertog, S., Havermann, F., Guo, S., Manola, I., Lejeune, Q., ..., &  Multi-Country Multi-City (MCC) Collaborative Research Network (2024).   Impacts of land-use & land-cover changes on temperature-related mortality | Kuwait | 2000-2016 | Longitudinal (Time-series design) | Quantitative  A two-stage approach: 1) quasi-Poisson regression with DLNMs (21 day lag); & 2) location-specific associations were pooled using a multivariate random meta-regression model. Using temperature from three fully coupled Earth system model simulations with both land use land cover change & no land use land cover change scenarios, authors estimated excess mortality due to non-optimal temperatures around the mid & end century. | Entire population included  Deaths: N/A | All events included (non-external deaths) | Outdoor average temperature | Meteorological data (nearest weather station used) | Daily | Single city | Non-external mortality | Daily | Ecological | A non-linear, somewhat U-shaped temperature-mortality relationship identified, but effect estimates not explicitly stated. | Year, seasonality, & day of the week. Temporally stable confounders adjusted for by design. | None | None  However, assessed land use land cover change variables at the sub-continental level (see 'Type of Analysis' for further information). |
| Yang, D., Hashizume, M., Tobías, A., Honda, Y., Roye, D., Oh, J., ..., & Chung, Y (2024).  Temporal change in minimum mortality temperature under changing climate: A multicountry multicommunity observational study spanning 1986–2015 | Kuwait | 2000-2016 | Longitudinal (Time-series design) | Quantitative  A two-stage approach: 1) used a quasi-Poisson regression with DLNM (21 day lag) was used to calculate the MMT & minimum mortality temperature percentile (MMTP) for each community for 5-year subperiods (i.e., 2001–2005, 2006–2010, & 2011–2015 for Kuwait); & 2) community-specific temporally varying estimates were pooled using mixed-effects meta-regressions to examine temporal changes in MMT & MMTP in the entire study population, by climate zone, geographical region, & country. | Entire population included  73,748 total deaths | All events included (all-cause or non-external deaths, not explicitly stated) | Outdoor average temperature | Not stated | Daily | Single city (extrapolated to the country level) | All cause or non-external deaths | Daily | Ecological | The temporal change in MMT & MMTP provided in a supplementary figure across subperiods only (no tabular values provided).   It appears that there was a slight increase in MMT identified, & no change for MMTP across the time periods. | Year, seasonality, & day of the week. Temporally stable confounders adjusted for by design. | None | None |
